# Oxygen-Based Nanocarriers to Modulate Tumor Hypoxia for Ameliorated Anti-Tumor Therapy: Fabrications, Properties, and Future Directions

**DOI:** 10.3389/fmolb.2021.683519

**Published:** 2021-07-01

**Authors:** Xianqiang Li, Yue Wu, Rui Zhang, Wei Bai, Tiantian Ye, Shujun Wang

**Affiliations:** Department of Pharmaceutics, College of Pharmacy, Shenyang Pharmaceutical University, Shenyang, China

**Keywords:** tumor hypoxia, nanoenzyme, oxygen, nanocarriers, tumor therapy

## Abstract

Over the past five years, oxygen-based nanocarriers (NCs) to boost anti-tumor therapy attracted tremendous attention from basic research and clinical practice. Indeed, tumor hypoxia, caused by elevated proliferative activity and dysfunctional vasculature, is directly responsible for the less effectiveness or ineffective of many conventional therapeutic modalities. Undeniably, oxygen-generating NCs and oxygen-carrying NCs can increase oxygen concentration in the hypoxic area of tumors and have also been shown to have the ability to decrease the expression of drug efflux pumps (e.g., P-gp); to increase uptake by tumor cells; to facilitate the generation of cytotoxic reactive oxide species (ROS); and to evoke systematic anti-tumor immune responses. However, there are still many challenges and limitations that need to be further improved. In this review, we first discussed the mechanisms of tumor hypoxia and how it severely restricts the therapeutic efficacy of clinical treatments. Then an up-to-date account of recent progress in the fabrications of oxygen-generating NCs and oxygen-carrying NCs are systematically introduced. The improved physicochemical and surface properties of hypoxia alleviating NCs for increasing the targeting ability to hypoxic cells are also elaborated with special attention to the latest nano-technologies. Finally, the future directions of these NCs, especially towards clinical translation, are proposed. Therefore, we expect to provide some valued enlightenments and proposals in engineering more effective oxygen-based NCs in this promising field in this comprehensive overview.

## Introduction

**FIGURE 1:**
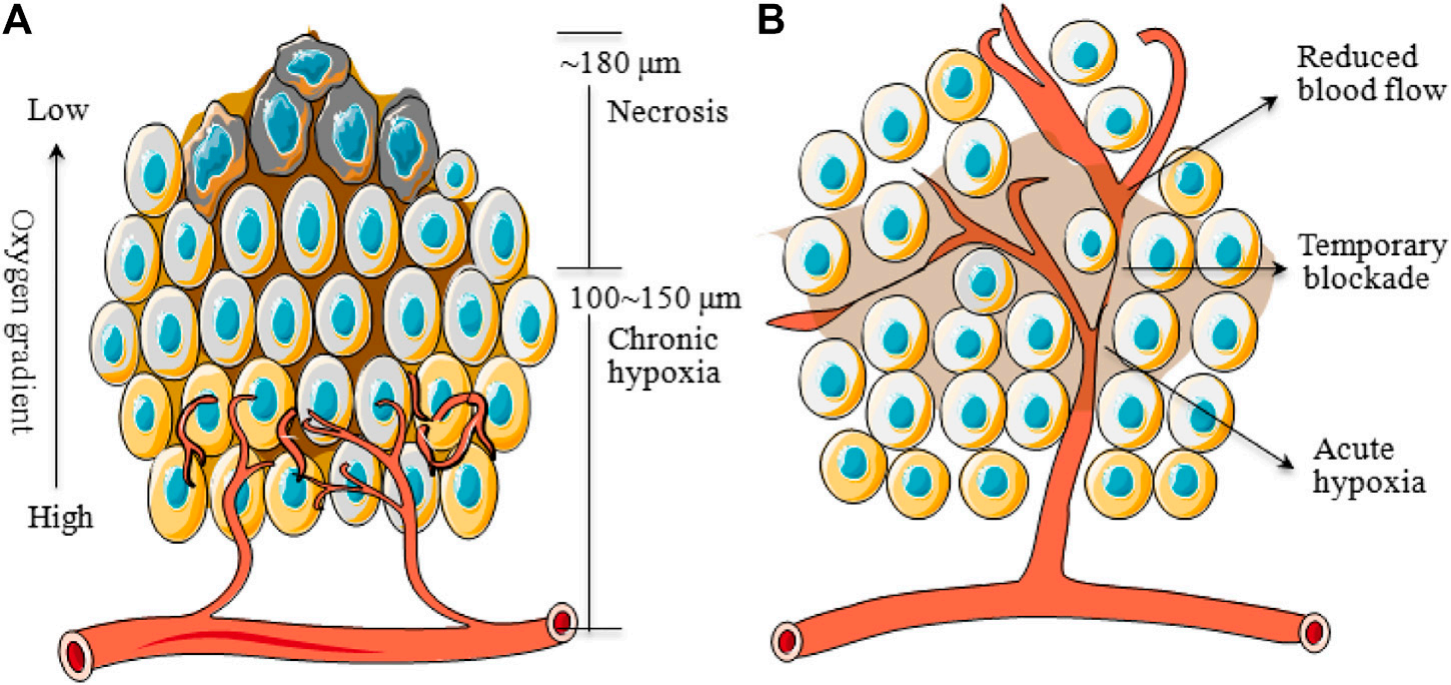
Schematic diagram of chronic and acute hypoxia of tumors. **(A)**: Regions of chronic hypoxia and necrosis are usually 100∼150 and ∼180 μm away from blood vessels. **(B)**: Areas of acute hypoxia can develop as a result of the temporary blockade or reduced flow in certain vessels.

The oxygen level of the human solid tumors is consistently less than the normal tissue that is so-called tumor hypoxia, which can be classified into two main categories. One is called chronic hypoxia or diffusion-limited hypoxia, which results from the imbalance between wild growth of tumor cells and new vasculature that would rapidly exhaust the oxygen in the original vessel. On the other hand, spatial distortion of tumor vasculature may lead to an increase in intercapillary distance that is beyond the diffusion capacity of oxygen (Patel and Sant, 2016). These hypoxic tumor cells are mainly distributed at a radius of about 100–150 μm from blood vessels to necrosis (Brown, 2007) (Figure 1A). And the necrosis would be observed with a radius of about 180 μm due to the complete anoxia (Wilson and Hay, 2011). The second type of tumor hypoxia, named acute hypoxia, perfusion-limited hypoxia, intermittent hypoxia, or cycling hypoxia, was caused by temporary obstruction or variable blood flow because of fluctuating tumor vessels (Figure 1B), which was first proposed by Brown (1979). In addition, tumor-associated or treatment-induced anemia can also bring tumor hypoxia through a decrease in oxygen transport capacity (Vaupel, 2004). Indeed, the oxygen partial pressure (pO_2_) in major human tumors is approximately 10 mmHg and in sharp contrast to that in respective normal tissue/organ is usually between 30 and 60 mmHg, with a difference of about 3∼20 times (Table 1). And an excellent review from Brown and Wilson (2004) demonstrated that the pO_2_ in all human tumors so far studied is lower than the normal tissue of origin and sometimes dramatically so.

**TABLE 1:** Typical pO_2_ of major tumors and respective normal tissue/organ.

Tissue/Organ	Tumor pO_2_ (mmHg)	Normal pO_2_ (mmHg)	Refs
Gliocyte	2.9∼4.9^a^	ND	Brown and Wilson (2004)
Lung	7.5^a^	38.5^a^	Brown and Wilson (2004)
Pancreas	2.7^a^	51.6^a^	Brown and Wilson (2004)
Prostate	2.4^a^	30.0^a^	Brown and Wilson (2004)
Skin	11.6^b^	40.5^b^	Sahu et al. (2020)
Brain	13^b^	35^b^	Vaupel et al. (2007)
Breast	10∼12^a^	50∼65^a^	Vaupel et al. (2007)
Head and neck	12.2∼14.7^a^	40.0∼51.2^a^	Brown and Wilson (2004), Vaupel et al. (2007)
Cervix	3.0∼5.0^a^	51.0^a^	Brown and Wilson (2004), Vaupel et al. (2007)
Vulva	10∼13^a^	ND	Vaupel et al. (2006)
Kidney	5.02 ± 1.12^b^	35.08 ± 2.41^b^	Lawrentschuk et al. (2011)
Liver	0.8^a^	4.0∼7.3^a^	Leary et al. (2002); Brooks et al. (2004)
Recta	19^a^	52^a^	Mattern et al. (1996)

a Note: Median tumor pO_2_

b Mean tumor pO_2_

ND: Not determined.

**FIGURE 2:**
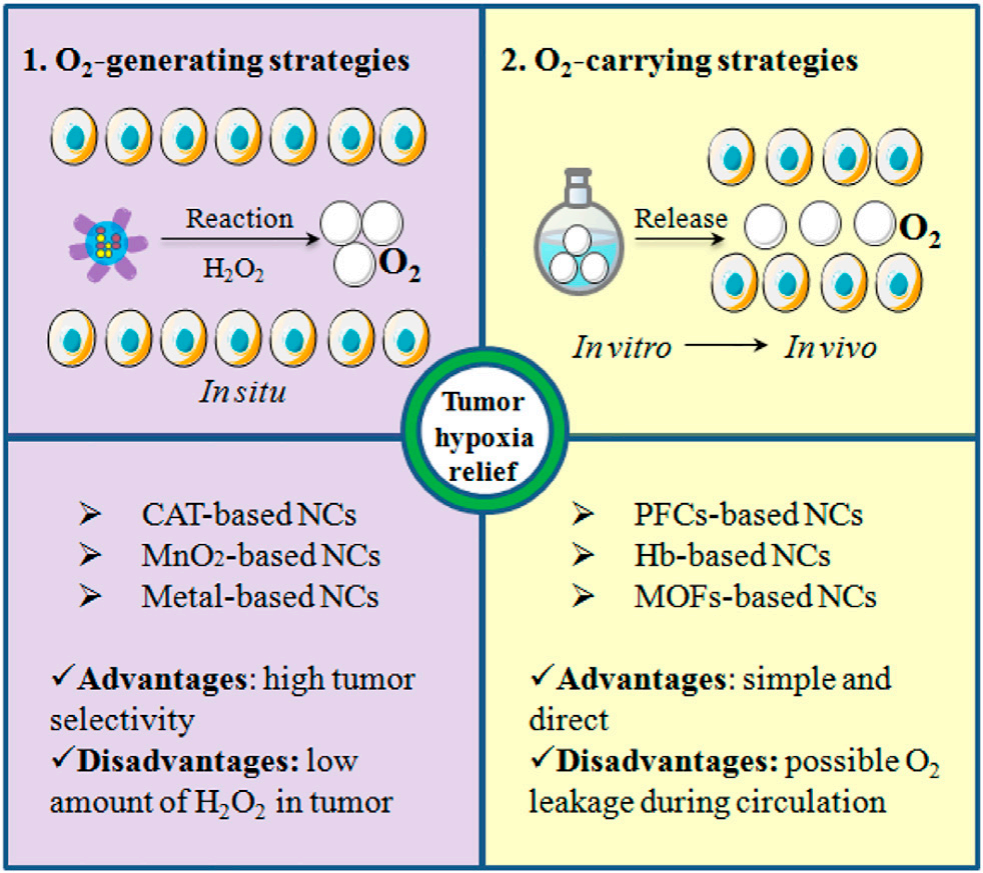
Schematic diagram of oxygen-generating strategies and oxygen-carrying strategies for tumor hypoxia relief: varieties, advantages, and disadvantages.

In recent years, a growing number of studies have proved that the hypoxic tumor microenvironment (TME) can seriously hinder the treatment outcomes of current therapeutic approaches involving oxygen as a key element for tumor destruction, mainly including anti-tumor drug-based chemotherapy (CMT) (Graham and Unger, 2018), the X-ray induced radiotherapy (RT) (Rey et al., 2017), photodynamic therapy (PDT) (Sun et al., 2020) and sonodynamic therapy (SDT) (Zhang N. et al., 2020), or even the latest anti-tumor immunotherapy (IMT) (Halpin-Veszeleiova and Hatfield, 2020). Consequently, replenishing oxygen to alleviate tumor hypoxia for enhancing therapeutic efficacy has attracted tremendous attention. Clinically, hyperbaric oxygen (HBO) therapy was introduced for this purpose. The patient is administered pure oxygen in a pressurized chamber to elevate the blood pO_2_ and thereby promoting oxygen transport to the hypoxic tumor tissue independent of hemoglobin. However, without a highly site-specific manner, HBO therapy can also lead to a high oxygen concentration in normal tissues which might cause serious side effects such as barotrauma, hyperoxic seizures and reactive oxygen species-mediated cytotoxicity (Moen and Stuhr, 2012). Additionally, due to the presence of acute hypoxia (Figure 1B), HBO therapy was not particularly successful (Brown and Wilson, 2004). To this end, many nanocarriers (NCs) are fabricated to overcome the inherent drawbacks of oxygen-based therapy via a high tumor-targeting property and thereby to provide an efficient, improved, and safer tumor treatment (Sahu et al., 2020). Moreover, these NCs also have many other outstanding features when the application in a tumor therapy such as a prolonged drug elimination half-life and improved drug stability in blood, controlled release, enhanced compliance with a patient's medication (Zhang A. et al., 2020). We classify these oxygen-based NCs into two major forms, including oxygen-generating strategies and oxygen-carrying tactics (Figure 2), based on the related publications. For the oxygen-generating strategies, it mainly uses oxygen generators such as catalase (CAT) (Song G. et al., 2016) or CAT-mimics (nanozymes) to decompose the over-expressed hydrogen peroxide (H_2_O_2_) to produce oxygen at the tumor cells. And the disadvantage is that the content of H_2_O_2_ inside the tumor is limited, which may not be able to meet the needs of treatment that requires a large amount of oxygen. By contrast, oxygen-carrying tactics are realized by physical dissolution or chemical conjugation in oxygen carriers including perfluorocarbons (PFCs) (Krafft, 2020), hemoglobin (Hb) (Yang J. et al., 2018) and metal-organic framework (MOF) (Xie et al., 2018) to directly release oxygen in tumor hypoxic regions. Although the oxygen-carrying NCs does not depend on the H_2_O_2_ level, it may release oxygen in the blood vessel prematurely.

**FIGURE 3:**
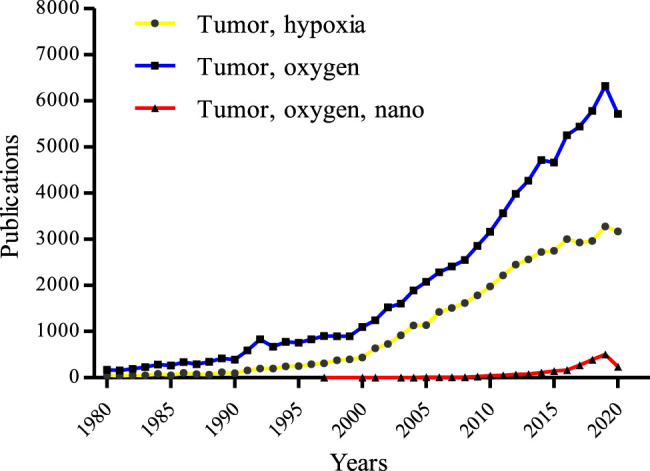
Tumor hypoxia-related publications over the 4 decades from the core collection of Web of Science. The blue line, yellow line, and red line represent data using the keywords of “tumor” and “oxygen”, “tumor” and “hypoxia”, and “tumor” and “oxygen”, and “nano”, respectively (update to Dec 31, 2020).

Remodeling of hypoxic TME with the help of NCs is of great importance to destroying a tumor (Liu et al., 2020). Although many researchers are focusing on designing NCs to circumvent hypoxia and encouraging results have been observed (Wang et al., 2020), most of these oxygen-based NCs are still in their infancy and few can be considered for clinical trials, let alone to become available for clinical use. The main obstacles that limit translation of these well-designed NCs from the lab to the clinic are some uncertain issues, such as safety, effectiveness, biocompatibility, etc., which need to be further evaluated. Besides, it is a rapidly evolving area of research with an increased understanding of complicated tumor biology, which drives us to continuously modify the design or synthesis of new NCs. The study of oxygen-based NCs is hundreds or even thousands of times less than the research on tumor hypoxia or tumor oxygen (Figure 3), suggesting that some more efficient and safer NCs capable of efficiently relieving tumor hypoxia to improved tumor therapy are needed. On this basis, an up-to-date account of the recent progress in the fabrications of hypoxia modulating NCs and their physicochemical and surface properties are systematically discussed followed by an introduction of the mechanisms of how tumor hypoxia affects the therapeutic effect. So, this review will deliver an in-depth analysis of NCs for tumor oxygenation, and it could also give valuable reference for future oxygen-based tumor therapy.

## Impact of Hypoxia on the Tumor Therapy

It has been well recognized that tumor hypoxia is a major barrier to the success of different types of therapies. A sustained hypoxic TME can cause a cascade of changes in gene transcription, protein expression, and metabolic processes, which not only facilitate the adaptation and growth of tumor cells in a hostile environment but also promote progression and metastases (Gilkes et al., 2014). In addition, hypoxia inducible factors (HIFs) will be activated and stabilized under a hypoxia condition, which in turn give rise to the transcription of some key genes involved in resistance of treatments (Semenza, 2012). In the clinic, it has been observed that many types of tumors, such as brain, head and neck, lung, stomach, ovarian, prostate, pancreas, colon, rectum, etc., are difficult to treat as well as have poor prognosis, directly or indirectly due to the hypoxia (Semenza, 2012; Dhani et al., 2015). The mechanisms of hypoxia that frustrates the therapeutic efficacy are complex and in part of which are discussed below.

### Chemotherapy

Driven by HIF-1α, drug efflux pumps such as P-glycoprotein (P-gp) are over-expressed, which can not only protect tumor cells by pumping cytotoxic chemotherapeutics to the extracellular spaces but also protect the molecular targets by transporting drugs into the lysosomal lumen from the cytoplasm (Harhaji-Trajkovic et al., 2009; Peng et al., 2014; Lee et al., 2015). Hypoxia-induced autophagy also has the capacity to reduce the drug concentration in tumor cells to decrease the slaying effect of CMT (Degenhardt et al., 2006). Many hypoxia-regulated microRNAs (miRNAs) can alter the sensitivity of drugs to various types of tumor cells through the different biological actions (Table 2). Besides, hypoxic cells are mostly located in the deep regions of tumor tissues (Figure 1A), which cannot be adequately exposed to CMT agents (Brown, 2007). Additionally, tumor cells with a low oxygen contend are found to have a markedly slower rate of proliferation, and however, most anti-tumor drugs are more effective against the rapidly dividing tumor cells than quiescent cells (Kondoh et al., 2010). Hypoxia is capable of selecting for tumor cells with low expression of apoptosis-related genes such as p53 genes, and resistance to apoptosis can be developed by inactivation of pro-apoptotic factors or by activation of anti-apoptotic factors (Roberts et al., 2009). Also, hypoxia can be a direct cause of CMT resistance because some drugs are oxygen-dependent and require oxygen to maximize cytotoxicity (Teicher, 1994).

**TABLE 2:** Hypoxic TME regulates miRNAs expression to induce CMT resistance.

miRNA	Regulation	Biological actions	Drugs	Tumors	Ref
miR-488	Up	Direct targeted bim	DOX	Osteosarcoma	Zhou et al. (2016)
miR-301b	Up	Suppressed bim expression	DOX	Lung cancer	Wu et al. (2016)
miR-497	Up	Targeted PDCD4	TMZ	Glioma	Lan et al. (2014)
miR-424	UP	Suppressed PDCD4 protein	DOX, EP	Colon cancer, melanoma	Zhang D. et al. (2014)
miR-421	Up	Targeted E-cadherin and caspase-3	CDDP	Gastric cancer	Ge et al. (2016)
miR-26a	Up	Protected response to mitochondrion	TMZ	Glioblastoma multiforme	Ge et al. (2018)
miR-301a	Up	Reduced TAp63 and PTEN level	GEM	Pancreatic cancer	Luo G. et al. (2018)
miR-338–3p	Down	Regulated HIF-1α	SOR	Hepatocarcinoma	Xu et al. (2014)
miR-338–5p	Down	Regulated feedback circuit	OX	Colorectal cancer	Xu K. et al. (2019)
miR-224–3p	Down	Regulated hypoxia induced autophagy	TMZ	Glioblastoma, astrocytoma	Huang et al. (2019)

### Radiotherapy

RT utilizes radioactive rays such as α, β, γ rays, electron beams or proton beams to destroy tumor cells by DNA damage. During the radiation, the high-energy photons provided by these rays can induce the production of DNA radicals (DNA·), which are unstable and can be quickly oxidized by oxygen, resulting in DNA double-strand breaks, thus leading to cell death. However, the hypoxic TME allows the cellular thiols such as glutathione to decrease the DNA radicals and significantly reduce the DNA double-strand breaks (Rey et al., 2017). Hence, adequate oxygen supply is an important guarantee for the efficacy of RT. As a result, hypoxic tumor cells are about 3 times more resistant to radiation than normoxic cells (Rockwell et al., 2009). The clinical data suggest that more hypoxic tumors in many types of human tumors such as head and neck cancers, prostatic carcinoma, melanoma, cervical cancer, etc., exhibit more radioresistant behaviour (Horsman and Overgaard, 2016).

### Photodynamic Therapy

PDT is a new promising therapeutic strategy, and its efficacy is critically dependent on oxygen. PDT uses tissue oxygen and photosensitizing molecules upon light irradiation to generate reactive oxygen species (ROS) such as singlet oxygen (^1^O_2_), superoxide (O_2_•‾), hydroxyl radical (HO•), etc., Subsequently, the unstable ROS reacts with components within the tumor cells such as DNA, proteins, and lipids, etc. to exert a cytotoxic effect, to destroy tumors (Zhang C. et al., 2020). During PDT treatment, the amount of cytotoxic ROS is directly determined by the oxygen concentration, thus the hypoxic cells are highly resistant to PDT. Meanwhile, PDT is a treatment process that continues to consume oxygen, which will exacerbate tumor hypoxia and further hamper the performance of PDT *in vivo*.

### Sonodynamic Therapy

SDT, as a novel noninvasive tumor ablation strategy, has the advantages of greater permeability, fewer side effects, and better patient compliance, when compared with PDT, but same to PDT that the tumor elimination ability is also challenged by insufficient oxygen supply. In SDT, ultrasound was employed to activate sonosensitizer from the ground state to an excited state, then the released energy is quickly transferred to the surrounding oxygen to form cytotoxic ROS, thus inducing the collapse of vacuoles and bringing irreversible damage to tumor cells (McEwan et al., 2015). However, similar to PDT, the generation of ROS will decrease significantly in a hypoxic TME, which will severely limit the therapeutic efficacy of SDT.

### Immunotherapy

IMT utilizes neoantigens, expressed by tumor cells, recognized by innate and adaptive immune cells such as T lymphocytes to induce anti-tumor immune responses, thereby eliminating tumors, which includes non-specific immune stimulation, adoptive T-cell transfer, immune-checkpoint blockade, and vaccination strategies (e.g., sipuleucel-T for prostate cancer). Although, this type of therapy does not involve the use of molecular oxygen, its clinical efficacy can also be frustrated by a hypoxic TME through changing the function of host immune cells, as often suggested in the literature (Noman et al., 2015). Indeed, lack of oxygen can directly inhibit the cytolytic activity of immune effectors such as natural killer (NK) and cytotoxic T cells (CTLs). Hypoxia reeducates immune cells toward an immunosuppressive phenotype, and influences maturation and function of the antigen-presenting cells (APCs) such as dendritic cells (DCs) (Noman et al., 2015) to diminish the activation of resting T cells. In hypoxia, an elevated level of HIF-1α can promote the production of various cytokines and growth factors such as vascular endothelial growth factor (VEGF) and stromal cell-derived factor 1α (SDF-1α). Then it will recruit, transform, and proliferate immunosuppressive cells to promote immune evasion, such as myeloid-derived suppressor cells (MDSCs), tumor-associated macrophages (TAMs) and T-regulatory (Treg) cells (Chouaib et al., 2017). Furthermore, driven by hypoxia, the expression of important immune checkpoint molecules is up-regulated, such as the cluster of differentiation 47 (CD47), programmed death-ligand 1 (PD-L1), and human leukocyte antigen G (HLA-G) (Li et al., 2018), which will also promote tumoral immune escape. Overall, IMT uses the power of the immune system to destroy tumor cells. On the contrary, hypoxia enhances the resistance of tumor cells to the immune system, which is a challenging for tumor IMT.

## Oxygen-Based Nanocarriers to Relieve Tumor Hypoxia

With rapid advances in biotechnology and nanomaterials, NCs drug delivery systems pave a new avenue for tumor therapy and diagnosis because of their ability to offer a great promise in improving specificity and efficiency. In view of the limitations of hypoxia on tumor treatment, a great effort on the fabrication of various NCs has been made to ameliorate hypoxic conditions. To provide a review of current developments in this promising field, we searched the PubMed, Web of Science, Google Scholar, Scopus, and Cochrane Central databases for related publications on oxygen-based NCs for boosted tumor therapy by using relevant keywords (tumor, hypoxia, and nano). 4,123 records and 616 of the closely related papers were screened for suitable studies in the past five years. In view of these related publications, the fabrications, characteristics, and treatment outcomes of the oxygen-generating NCs and oxygen-carrying NCs were systematically discussed (Table 3 and Table 4).

**TABLE 3:** Oxygen generating NCs to modulate tumor hypoxia for enhanced anti-tumor therapy.

Design	Size (nm)	Oxygenation efficacy	Therapies	*In vivo* TGI	Mechanisms	Ref
CAT-based NCs						
TaOx@CAT NPs functionalized with PEG	∼119	Hypoxia positive areas reduced from 65 to 15%	RT	>95%	Increased radiation-induced DNA damage	Song G. et al. (2016)
Liposomes containing CAT and cisplatin-prodrug	∼100	Hypoxia positive areas decreased from76.9 to 11.45%	CMT-RT	∼85%	Induced high level of DNA damage	Zhang R. et al. (2017)
Liposomes encapsulating CAT, MBDP and DOX	∼122	Hypoxia positive regions reduced from 20 to 12%	PDT-CMT	>95%	Facilitated ^1^O_2_ production, improved immune response	Shi et al. (2020)
HSA-based NPs loaded CAT, PTX, and Ce6	∼100	Hypoxia positive areas decreased from 32 to 7%	PDT-CMT	∼80%	Increased the production of ^1^O_2_	Chen Q. et al. (2017)
PLGA-based NPs loaded CAT, MB and BHQ-3	∼205	The intracellular O_2_ levels increased gradually *in vitro*	PDT	∼100%	Induced the formation of cytotoxic ^1^O_2_	Chen et al. (2015)
HA-based NPs loaded CAT and Ce6	∼233	Retained more than 90% of CAT enzymatic activity *in vitro*	PDT	∼85%	Enhanced the production of ^1^O_2_	Phua et al. (2019)
Cell membrane loaded ZIF-8, CAT and DOX	∼130	Mixing it with 10 × 10^6^ M H_2_O_2_ can increase 15 mg/L O_2_ in 400 s	CMT-IMT	∼100%	Reduced the expression of HIF-α and PD-L1	Zou et al. (2018)
MnO_2_-based NCs						
MnO_2_ nanosheets anchored upconversion nanoprobes	100∼200	Enhanced tumor vascular saturated O_2_ about 6 times	PDT-RT	>95%	Boosted the kinetics of ^1^O_2_ generation	Fan et al. (2015)
NPs composed of albumin and MnO_2_	∼50	Tumor hypoxic area decreased by 24%, 45% within 30 min, 60 min	RT	∼70%	Increased DNA double strand breaks	Prasad et al. (2014)
HA-coated MnO_2_ NPs loaded DOX	∼203	The percentage of hypoxia areas decreased by 64.5%	CMT-IMT	∼50%	Primed TAMs toward m1-like phenotype	Song M. et al. (2016)
HA-modified NPs loaded MnO_2_ and ICG	∼240	Oxygen content in the tumor elevated about 2.25 times	PDT	∼100%	Facilitated ^1^O_2_ production, reducing HIF-α expression	Gao et al. (2017)
HSA-coated MnO_2_ NPs loaded Ce6 and Pt (IV)	∼50	Hypoxia positive areas decreased from 33 to 9%	PDT-CMT	∼90%	Enhanced drug uptake and ^1^O_2_ production	Chen et al. (2016)
PEGylated MnO_2_ NPs containing Ce6	∼100	Hypoxia positive areas decreased from 36 to 12% within 12 h and to 4% within 24 h	PDT	∼80%	Increased the production of ^1^O_2_	Zhu et al. (2016)
MnO_2_ based NPs containing of WS_2_, Fe_3_O_4_, SiO_2_ and PEG	∼182	Hypoxia positive areas decreased from 43 to 10% within 12 h and to 3% within 24 h	PTT-RT	>90%	Generated a high level of DNA damage	Yang G. et al. (2018)
BSA-Au-MnO_2_ composite NPs	∼60	Hypoxia positive areas decreased from 20 to 2%	RT	∼90%	Improved the susceptibility of tumor cells to X-ray	Chen J. et al. (2017)
PEGylated hollow MnO_2_ nanoshells containing Ce6 and DOX	∼15	Hypoxia positive areas decreased from 41 to 10% within 6 h and to 6% within 12 h	CMT-PDT	>80%	Reversed the immunosuppressive TME	Yang et al. (2017)
Radionuclide^131^I labeled HSA-bound MnO_2_ NPs	∼40	Hypoxia positive areas decreased from 35 to 10%	RT	∼90%	Promoted DNA damages	Tian et al. (2017)
RBC membrane composed MnO_2_, PB, and DOX	∼67	Relieved tumor hypoxia situation	PTT-CMT	∼100%	Enhanced anti-tumor drug uptake	Peng et al. (2017)
UCNPs@TiO_2_@MnO_2_ core/shell/sheet NCs	∼80	Increased the dissolved O_2_ about 30 mg/L within 20 min *in vitro*	PDT	∼100%	Increased the production of ^1^O_2_	Zhang C. et al. (201
MnO_2_-based hybrid semiconducting NPs	40∼76	Triggered about 2.5 mg/L O_2_ in the H_2_O_2_ solution within 10 min	PDT	>90%	Generated 2.68-fold more ^1^O_2_ at hypoxic TME	Zhu H. et al. (2018)
MnO_2_ functionalized albumin bound PTX NPs	∼140	Tumor O_2_ concentration was 50 μM after intratumoral injection	CMT-RT	96.57%	Stabilized DNA damages	Meng et al. (2018)
MnO_2_ coated SiO_2_-MB nanocomposites	∼300	Elevation of O_2_ concentration in the H_2_O_2_ solution within 4 min	PDT	>90%	Promoted the kinetics of ^1^O_2_ generation	Ma et al. (2017)
Core-shell gold nanocage coated with MnO_2_	∼91	The percentage of hypoxia areas dramatically disappeared	PDT-IMT	∼100%	Promoted ^1^O_2_ production, elicited immune cell death	Liang et al. (2018)
DSPE-PEG 2000 modified MnO_2_ based NPs	∼110	Improved the dissolved O_2_ about 20 mg/L within 10 min	PDT	>95%	Increased the production of ^1^O_2_	Jia et al. (2018)
MnO_2_ based NPs loaded DOX, g-C_3_N_4_ and F127	∼78	Triggered about 16 mg/L O_2_ in the H_2_O_2_ solution within 3 min	CMT-PDT	∼100%	Increased ^1^O_2_ generation and allayed DOX resistance	Zhang W. et al. (2018)
MnO_2_-hollow mesoporous organsilica NPs	∼90	Improved the dissolved O_2_ about 7 mg/L within 1 min	SDT	∼96%	Promoted ^1^O_2_ production, reduced HIF-α expression	Zhu P. et al. (2018)
MnO_2_-PEGylated black phosphorous nanosheet	∼120	The percentage of hypoxia areas were decreased	PDT	∼100%	Enhanced ^1^O_2_ generation, reduced HIF-α expression	Liu et al. (2019)
MnO_2_-loaded, BSA and PEG co-modified mesoporous CaSiO3 NPs	∼110	The O_2_ saturation inside tumors increased from 3 to 20% within 24 h	CMT	∼95%	Increased chemodrug uptake by tumor cells	Guo et al. (2020)
Pt based NCs						
Pt based NCs containing of MPDA, BSA, Ce6 and DOX	∼140	The dissolved O_2_ increased more than 20 mg/L within 120 s *in vitro*	PDT-CMT	∼100%	Increased the production of ^1^O_2_	Hu et al. (2019)
Pt based hybrid core-shell NCs	∼200	Enhanced the dissolved O_2_ about 50 mg/L within 25 min	PDT	>90%	Enhanced the production of the cytotoxic ROS	Xiao-Shuang et al. (2018)
Pt and Pd nanoplates modified with PEG and conjugated with Ce6	∼30	Effectively decomposed intracellular H_2_O_2_ into oxygen *in vitro* and *in vivo*	PDT	∼100%	Promoted the generation of the cytotoxic ROS	Wei et al. (2018)
Pt NPs decorated MOFs	∼90	Promoted H_2_O_2_ to O_2_ conversion by the presence of Pt NPs *in vivo*	PDT	>90%	Increased the production of ^1^O_2_	Zhang Y. et al. (2018)
PEGylated porous Pt NPs	∼116	Well relieved the tumor cell hypoxia situation	RT	∼90%	Decreased RT resistance by promoting O_2_ generation	Li Y. et al. (2019)
Fe-based NCs						
Fe-TBP nanorice co-assembly of MOFs	∼100	Effectively catalyzed H_2_O_2_ into oxygen *in vitro* and *in vivo*	PDT-IMT	>90%	Improved a-PD-L1 therapy increased ^1^O_2_ generation	Lan et al. (2018)
Mn-Fe NP anchored mesoporous silica NPs	∼56	Tumor O_2_ saturation increased from 1.5 ± 0.2% to 12.6 ± 1.9%	PDT	>90%	Improved ROS generation	Kim et al. (2017)
PEG modified liposome loaded holo-Lf and DOX	∼180	Hypoxia positive regions decreased from 60.2 to 17.3%	RT-CMT	∼100%	Strengthened the cell DNA damage	Zhang et al. (2019b)
Ce-based NCs						
Lanthanide ion-doped mesoporous hollow cerium oxide upconversion NPs	∼160	Tumor blood oxygen saturation increased from 6.9 to 19.2% in 100 min	PDT-CMT	∼90%	Enhanced the drug uptake, potentiated ROS-mediated cytotoxicity	Yao et al. (2018)
Ce-based NCs comprising of DOX	∼48	The quantity of oxygen produced reaches 2.06 mg/L and 1.32 mg/L in H_2_O_2_ solution within 30 min	PDT-CMT	∼90%	Increased the DOX uptake, boosted ROS-mediated cytotoxicity	Jia et al. (2019)

**TABLE 4:** Oxygen carrying NCs to modulate tumor hypoxia for enhanced anti-tumor therapy.

Design	Size (nm)	Oxygenation efficacy	Therapies	*In vivo* TGI	Mechanisms	Ref
PFCs-based NCs						
PFC-loaded hollow Fe_3_O_4_ magnetic nanoplatform	∼13	Hypoxia positive areas decreased from ∼78 to ∼18%	CMT	∼85%	Alleviated the hypoxia induced CMT resistance	Zhou et al. (2019)
PFTBA@HSA NPs	150∼200	Enhanced tumor relative oxygen pressure from 90 to 220%	CMT	>80%	Enhanced the hypoxia associated cytotoxic	Zhou et al. (2018b)
Albumin-based NPs loaded PFC and HSA	∼150	Relieved short-term and long-term tumor hypoxia	RT	∼90%	Promoted radiation-induced cell damage	Zhou et al. (2018a)
PFP-based PLGA NPs loaded ICG and PTX	∼186	Relieved tumor cell hypoxia situation	PDT-SDT, CMT	∼80%	Decreased the expression of MDR-1	Chen S. et al. (2018)
PFOB-based nanoemulsion	∼197	The oxygen level increased from 62.8 to 83.5%	CMT	∼75%	Reduced hypoxia-induced CMT resistance	Song et al. (2019)
Hb-based NCs						
Hb loaded nanoliposome loaded DOX	∼151	Effectively alleviated hypoxic state both *in vitro* and *in vivo*	CMT	∼70%	Enhanced ROS-mediated cytotoxicity	Yang J. et al. (2018)
Hb and albumin NPs loaded DOX and Ce6	∼30	Modulated tumor hypoxia by donating bound oxygen deep in the tumor	CMT-PDT	89.5%	Decreased the expression of P-gp and MDR-1, increased the production of ^1^O_2_	Luo Z. et al. (2018)
Hb connected with Ce6 NPs with SOR	∼175	Well relieved tumor hypoxia situation	PDT	>90%	Boosted ROS generation and enhanced the ferroptosis	Xu et al. (2020)
ZnF_16_ Pc-loaded ferritin RBC	∼7,000	Provided O_2_ to enable sustained ^1^O_2_ production under hypoxia	PDT	76.7%	Continuous increased the production of ^1^O_2_	Tang et al. (2016)
RBC microcarriers	∼7,000	The oxygenated Hb percentage *in vivo* increased from 7 to 30%	PDT	>90%	Effectively promoted the generation of ^1^O_2_	Wang et al. (2017)
MOFs-based NCs						
UiO-66 MOF conjugated with ICG and coated with RBC membranes	∼65	Obviously elevated oxygen level in tumors	PDT	∼100%	Enhanced the production of the cytotoxic ROS	Gao et al. (2018)
Mesoporous silica coated ZIF-90	∼120	Released a large amount of O_2_ in an acidic TME	PDT-CMT	∼90%	Increased cytotoxic of DOX and ^1^O_2_ generation	Xie et al. (2018)
Increasing intratumoral blood flow						
MnSe@Bi_2_Se_3_ NPs	∼140	Reduced the hypoxic level in almost the whole tumor	RT	∼90%	Improved blood flow into tumors	Song et al. (2015)
MoS_2_ based NPs	∼21	Relieved tumor hypoxia through hyperthermia	PTT-RT	∼95%	Enhanced tumor blood flow under hyperthermia	Wang J. et al. (2016)
Decreasing intratumoral oxygen consumption						
Met-based PEGylated liposomes loaded Ce6	∼110	Oxyhemoglobin saturation increased from 14.6 to 30.1%	PDT	>75%	Decreased oxygen consumption to promote ^1^O_2_ generation	Song et al. (2017)
Met and W_18_O_49_ NPs co-loaded into platelet membranes	∼115	Almost no detectable hypoxia signal in tumors	PDT-PTT	∼90%	Decreased oxygen consumption to enhance ROS production	Zuo et al. (2018)

### Oxygen-Generating Strategies

**FIGURE 4:**
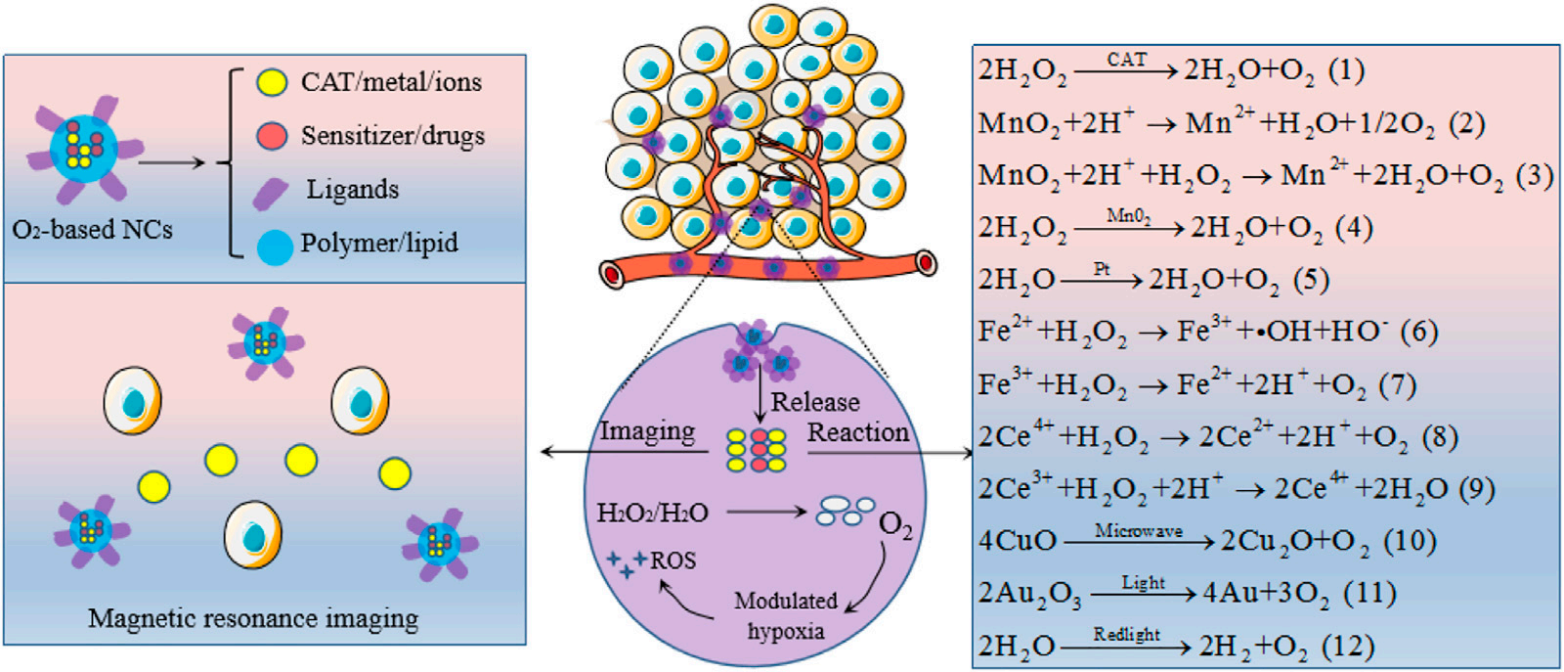
Oxygen-generating NCs to modulate tumor hypoxia and to endow magnetic resonance imaging. The specific mechanisms of oxygen generation are represented by chemical equations.

Human solid tumor cells generate high levels of H_2_O_2_ as a second messenger to induce resistance to therapy and enhance tumor malignant progression. It has been reported that intracellular H_2_O_2_ concentration can be as high as 50∼100 μM in the tumor hypoxia region (Castaldo et al., 2019), while 1∼10 nM (Stone and Yang, 2006) or 1∼700 nM (Sies, 2017) in normal cells by different estimate. The elevated endogenous H_2_O_2_ mainly generates from aberrant cellular metabolism in the electronic respiratory chain, which is produced by the action of oxygen-derived species superoxide anion through the superoxide dismutase enzymolysis (López-Lázaro, 2007). Therefore, the hypoxic TME could be readily modulated by decomposing the endogenous H_2_O_2_ into O_2_ with various catalytic NCs (Figure 4).

#### Catalase-Based Nanocarriers

CAT, an enzyme to rapidly decompose H_2_O_2_ into H_2_O and O_2_, as shown in equation (1), but the expression of tumor cells is significantly down regulated when compared to normal tissues (Glorieux et al., 2015). Hence, specific targeting of exogenous CAT to tumor hypoxic zone has been widely explored in recent years. However, protease-induced degradation of CAT may severely restrict its *in vivo* application due to a complicated physiological environment (Song G. et al., 2016). With this regard, various CAT-based NCs have been developed to retain its catalytic activity during the tumor-targeted delivery process. Liposomes have a high capacity to encapsulate water-soluble CAT in its inner water phase. For instance, Zhang et al. constituted CAT and cisplatin-prodrug-encapsulated liposomes, and demonstrated that the enzyme activity of CAT within liposome was well protected. By semi-quantitative analysis of hypoxia positive signals in tumor slices, they found that the percentages of hypoxia regions decreased from 76.9 to 11.45% (Zhang R. et al., 2017). Similarly, Peng group developed a unique liposome by encapsulated CAT, lyso-targeted NIR photosensitizer and doxorubicin (DOX) to catalyze intratumoral high-expressed H_2_O_2_. They suggested the tumor hypoxic areas were significantly reduced, which not only can facilitate the ^1^O_2_ production but also reverse immunosuppressive TME (Shi et al., 2020). Nanoparticles (NPs), another promising and effective drug delivery system, also have a strong ability to load CAT into the inside of NPs, thus can well protect the catalytic activity and stability of CAT in the circulatory system. For example, CAT entrapped in human serum albumin (HSA) NPs could maintain ∼70% of its initial enzymatic activity after incubation with protease K for 24 h (Chen Q. et al., 2017). Even better, CAT encapsulated hollow tantalum oxide (TaOx) NPs have the ability to only lose less than 5% of the enzymatic activity. Making use of PLGA approved for clinical use by FDA, Chen et al. (2015) designed PLGA-based NPs by loaded CAT, methylene blue (MB), and black hole quencher-3 (BHQ-3) against the hypoxic tumor cells. *In vivo* studies confirmed that the CAT activity was well retained and thus intracellular oxygen level was gradually increased. In addition to PLGA, hyaluronic acid (HA) is another excellent drug carrier, which has been extensively researched to load anti-tumor drugs for tumor targeting. Phua et al. (2019) fabricated CAT encapsulated HA NPs and suggested that the CAT enzymatic activity was retained more than 90%. Zou et al. (2018) reported a multifunctional biomimetic core-shell nanoplatform with loaded CAT, zeolitic imidazolate framework 8 (ZIF-8) and DOX, which can increase 15 mg/L oxygen in 400 s by mixing it (CAT: 1370 U/mg) with 10 × 10^6^ M H_2_O_2_, illustrating that such a NC did not inactivate CAT to decompose H_2_O_2_ for oxygen production.

#### Manganese Dioxide-Based Nanocarriers

Manganese dioxide (MnO_2_) has a strong capacity to generate oxygen for hypoxic tumor cells on one hand by reacting with H_2_O_2_ and excess hydrogen ions and on other hand has an enzyme-like activity to catalyze H_2_O_2_, as shown in equation (2), (3) and (4). It could be decomposed to water-soluble Mn^2+^ ions, thus leading to the dissolution of these NCs for therapy (Fan et al., 2015). Besides, Mn^2+^ ions could also be utilized for magnetic resonance imaging (MRI). As such, MnO_2_-based NCs to date are the most common and featured CAT-mimic for tumor oxygenation.

The use of MnO_2_ for modulated tumor hypoxia was first reported by Prasad et al., in 2014. They engineered multifunctional and colloidally stable bioinorganic NPs composed of polyelectrolyte albumin complex and MnO_2_ NPs (A-MnO_2_ NPs). *In vivo* studies, they found that tumors treated with such a MnO_2_-based NC for 30 min, 60 min, or 24 h showed 24, 45, and 37% less tissue hypoxia, respectively. And the same tumors also showed a 19, 21, and 10% decrease in the expression of HIF-α, and 7, 65, and 65% decrease in the expression of VEGF, after 30 min, 60 min, and 24 h, respectively (Prasad et al., 2014).

In 2015, Gordijo et al. (2015) constructed two different hybrid NPs by embedding polyelectrolyte-MnO_2_ (PMD) in hydrophilic terpolymer/protein-MnO_2_ (TMD) or hydrophobic polymer/lipid-MnO_2_ (LMD) matrices. They employed fluorescein isothiocyanate-pimonidazole and Alexa 594 conjugated antibody to detect tumor hypoxia and the expression of HIF-1α, and the control tumors were observed to be very hypoxic (90% positive area) and high expression of HIF-1α (70% positive area). Following treatment with intratumoral injection, the fast-acting TMD and slow-acting LMD NPs decreased hypoxia positive area by about 70 and 33% within 0.5 h, and led to similar decreases in HIF-1α, of around 40 and 60% after 2 and 4 h, respectively. However, the tumor hypoxia and expression of HIF-1α did not show significant changes when mice treated with i. v. injection of TMD NPs, while LMD NPs decreased tumor hypoxia by 30 and 45% at 2 and 4 h post i. v. injection and reduced HIF-1α by 55% within 4 h, respectively. In another attempt, MnO_2_ nanosheets anchored with upconversion nanoprobes (UCSMs) were developed by Shi group to overcome hypoxia. Results from their study suggested that such UCSMs can not only significantly increase the oxygenated/deoxygenated hemoglobin but also enhance tumor vascular saturated oxygen by about 7%. Meanwhile, the expression of HIF-1α also remarkably inhibited. In addition to relieving hypoxia, much stronger yellow luminescence can be detected surrounding the nucleus after incubation with hypoxic murine breast cancer cells for 8 and 20 h, which means that the upconversion luminescent imaging and tumor therapy could be simultaneously achieved (Fan et al., 2015). However, the doses of these two kinds of MnO_2_-based NCs delivered into solid tumors were all limited by i. v. injection, hence, more effective strategies such as proper surface modification would be adopted to enhance tumor accumulation.

In 2016, there was two publications that utilized HA modification (Song M. et al., 2016; Gao et al., 2017), one paper used HSA coating (Chen et al., 2016), and one report employed PEGylation (Zhu et al., 2016) to construct intravenous MnO_2_-based NCs for tumor oxygenation, respectively. Owing to improved surface properties, tumor homing of these NCs by i. v. injection was greatly enhanced, thus the intratumoral hypoxia can be alleviated to a great extent. As an example, HA modified MnO_2_-based NPs designed by Gao et al. (2017) was found to be a high tumor accumulation. The tumor-muscle ratio was 4.03 ± 0.36 for fluorescent imaging and 2.93 ± 0.13 for photoacoustic imaging. After i. v. injection, the oxygen content in the tumor was elevated 2.25 ± 0.07 times. Furthermore, HA also has immunotoxicological effect on macrophages, which can activate macrophages and increase the generation of endogenous ROS. Taking this advantage, HA-coated MnO_2_ NP was fabricated by Song M. et al. (2016) to facilitate the transformation of anti-tumor M2 macrophages to the M1 phenotype. Their results demonstrated that the tumor hypoxia was further relieved. The HSA coated and PEGylated MnO_2_-based NCs from the Liu group were clearly found to be highly effective to decompose tumor over-expressed H_2_O_2_ into oxygen via systemic administration, and the percentage of hypoxia positive areas decreased significantly by 2∼4 times (Chen et al., 2016; Zhu et al., 2016).

More researchers had invested in the fabrications of MnO_2_-based NCs to ameliorate anti-tumor therapy in the year of 2017 with nine publications. Of note, they also found that the Mn^2+^ ions released from the MnO_2_ NPs can serve as a diagnostic agent to enable the identification of tumor regions by MRI during therapy. In a study from Liu group, tungsten disulfide (WS_2_) nanoflakes with their surface adsorbed with iron oxide NPs via self-assembly are coated with silica and then with MnO_2_, on to which PEG is attached. *In vivo* evidence has demonstrated that the hypoxia positive areas were decreased from 43 to 10% within 12 h and to 3% within 24 h. After oxygenation, a high level of DNA damage has been generated, which offers remarkable benefits for PTT together with RT. At the same time, strong photoacoustic signals were observed in tumors, suggesting that such a design could be used as a pH-responsive MRI probe for tumor diagnosis and detection (Yang G. et al., 2018). For the same aim, their group also engineered a size-changeable, BSA modified, Au nanoclusters and MnO_2_ composite NPs, which not only can penetrate the dense extracellular matrix to reverse tumor hypoxia and show strong red fluorescence to facilitate imaging, but also act as a radio-sensitizer by absorbing and depositing X-ray energy within tumors to enhance RT (Chen J. et al., 2017). With the same objective to overcome hypoxia-associated therapy resistance and enable tumor-specific imaging, Yang et al. (2017) constructed intelligent biodegradable hollow MnO_2_ nanoshells modified with PEG and co-loaded with a photodynamic agent and a chemodrug. The as-prepared NCs would be effectively dissociated under acidic TME pH to catalyze tumor endogenous H_2_O_2_ to generate oxygen, which decreased hypoxia positive area from 41 to 10% within 6 h and to 6% within 12 h and, in the meantime, particularly useful for tumor-specific MR imaging. In a similar endeavor, HSA-templated MnO_2_ NP with suitable sizes was successfully developed by Tian et al. (2017), which was found to be dramatically improved tumor oxygenation upon systemic administration. In a separate undertaking, Peng et al. (2017) constructed a NC by loading Prussian blue (PB)/MnO_2_ as an oxygen precursor or catalyzer for H_2_O_2_ activation, and a red blood cell (RBC) membrane was used to increase the loading capacity of DOX and prolong the circulation time *in vivo*. Mixing such a MnO_2_-based NC with 10 × 10^6^ M H_2_O_2_ can increase 15.3 mg/L oxygen within 1 min *in vitro*, and the generated O_2_ in tumor site can disrupt the RBC coated on the surface of PB/MnO_2_, which increases the ability of DOX to kill tumor cells. Zhang C. et al. (2017) constructed a versatile nanoplatform, upconversion NPs@TiO_2_@MnO_2_ core/shell/sheet nanocomposites to address insufficient oxygen supply, inefficient ROS production, and low penetration depth of light during the PDT process. Following i. v. injections, all these drawbacks have been well solved by generating oxygen *in situ*, increasing the concentration of ^1^O_2_ and hydroxyl radical (•OH) *via* water-splitting, and employing 980 nm NIR light to enhance penetration depth. Also, the decomposed Mn^2+^ can be utilized for further upconversion luminescence and MRI in tumor site. Similarly, Zhu H. et al. (2018) designed hybrid core-shell NPs coated with semiconducting polymer NPs and MnO_2_ nanosheets via a one-pot surface growth reaction, which can generate 2.68-fold more ^1^O_2_ at hypoxic and acidic conditions under NIR laser irradiation and also can serve as the NIR fluorescence imaging. Lastly, MnO_2_ functionalized albumin-bound paclitaxel NPs and MnO_2_ coated SiO_2_-MB nanocomposites were engineered by Meng et al. (2018) and Ma et al. (2017), which all demonstrated that the oxygenic MnO_2_-based NCs have a good tumor hypoxia relief ability and were capable of *in vivo* MRI selectively in response to over-expressed acidic H_2_O_2_.

In 2018, three scientists synthesized MnO_2_ related NCs to boost PDT effect including core-shell gold nanocage@MnO_2_ NPs (Liang et al., 2018), DSPE-PEG 2000 modified MnO_2_ NPs (Jia et al., 2018), and MnO_2_ NPs loaded DOX, g-C_3_N_4_ and F127 (Zhang W. et al., 2018), as well as one study from Zhu P. et al. (2018) had fabricated MnO_2_-hollow mesoporous organsilica NPs to improve the SDT therapy efficacy. Subsequently, Liu et al. (2019) constructed a MnO_2_-PEGylated black phosphorous nanosheet via the electrostatic assembly for PDT, which once again clearly proves that the MnO_2_-based NCs could dramatically decrease the percentage of tumor hypoxia areas. In 2020, Guo et al. (2020) developed a MnO_2_-loaded, BSA, and PEG co-modified mesoporous CaSiO_3_ NP to modulate hypoxic TME for effective cancer therapy. Results from their study suggested that the hypoxic conditions were remarkably improved by reacting with endogenous H_2_O_2_ and T1-weighted MRI could be activated owing to the Mn^2+^ released from MnO_2_ NPs in TME, which offered a diagnostic strategy for the real-time monitoring of therapeutic effects. Details of the design, size, oxygenation efficacy, therapies, *in vivo* tumor growth inhibition, and mechanisms of individual NCs are given in Table 3.

#### Metal-Based Nanocarriers

Platinum (Pt) NPs, a kind of well-known nanozyme, exhibits excellent CAT-like activities as shown in equation (5). Shi research group constructed a multi-functional nanoplatform with composing of mesoporous polydopamine, Pt NPs, BSA, Ce6 and DOX (M-Pt-BCD) to overcome tumor hypoxia. After mixing it with H_2_O_2_ solution, the dissolved oxygen can be increased by more than 20 mg/L within 120 s. The production of ^1^O_2_ was remarkably enhanced as compared with the control group of water, H_2_O_2_, and M-BCD in a tumor simulation TME, so the key component for catalase capacity was Pt NPs. The increased synergistic effect of CMT and PDT *in vivo* also proves that Pt NPs can be used as an effective nanozyme to relieve tumor hypoxia (Hu et al., 2019). As a catalyst, Pt NPs could also be incorporated into a hybrid core-shell nanoplatform to work like a nanofactory, which can provide the necessary oxygen and the lethal ROS for destroying tumors by decomposing the endogenous H_2_O_2_ (Xiao-Shuang et al., 2018). In an interesting study from Zheng group, Pt and metal Palladium (Pd) were designed as a bimetallic nanoplates, then further modified with bifunctional PEG and covalently conjugated with the photosensitizer Ce6 to form a nanosheet-shaped photosensitizer. Both *in vitro* and *in vivo* results indicated that such a Pt based NCs effectively decomposed intracellular H_2_O_2_ into oxygen, resulting in a significantly boosted PDT efficacy (Wei et al., 2018). In another attempt, Zhang Y. et al. (2018) decorated Pt NPs onto photosensitizer integrated MOFs to form a versatile nanosystem with high CAT-like activity. As they expected, such a Pt based nanoplatform can promote the formation of ^1^O_2_ in a hypoxic tumor site *via* H_2_O_2_-activated evolvement of oxygen, which can produce more serious damage to tumor cells. In addition to the catalytic oxygen generation capability, Pt NPs are high Z-element material that can be used as a RT sensitizer. It is reported that Porous Pt NPs can not only address the hypoxic TME issue but are also capable of overcoming the RT resistance in a non-small-cell lung cancer model (Li Y. et al., 2019). Besides the examples given here, continuous undertakings focus on optimizing the fabrications of Pt-based NCs by combining with other nanostructures such as Pt/PCN-224(M)-MOF (Chen Y. Z. et al., 2017), ferritin-Pt NPs (Fan et al., 2011), Pt and CoOx co-catalysts (Zhang J. et al., 2017) to yield diverse functionality to further alleviate tumor hypoxia.

Recent research has reported that iron (Fe) associated NPs could initiate a Fenton-like reaction (as described in equation (6) and (7), also named Fenton chemistry) to catalyze oxygen production from endogenous H_2_O_2_ under hypoxic TME. Lan et al. (2018) reported a nanoscale MOF, Fe-tetra (p-benzoato) porphyrin (TBP), as a novel nano-photosensitizer, to overcome tumor hypoxia based on the Fenton reaction. When irradiated under hypoxic conditions, the competency of Fe-TBP in elevating oxygen generation by Fe_3_O clusters improved the tumor hypoxia and in turn significantly increased the lethal ^1^O_2_ by photoexcited porphyrins. Similarly, Hyeon research group designed biocompatible manganese ferrite NP-anchored mesoporous silica NPs (MFMSNs) to overturn hypoxia based on the Fenton reaction. As anticipated, after i. v. injection of MFMSNs to mice models, the O_2_ saturation inside tumor zones increased from 1.5 ± 0.2% to 12.6 ± 1.9% 24 h and HIF-1α expression was also significantly down-regulated. More interestingly, MFMSNs were not consumed during the Fenton reaction, which could continuously produce O_2_ by decomposing H_2_O_2_ (Kim et al., 2017). Besides, holo-lactoferrin (holo-Lf), a natural protein, can not only act as a potential ligand of the transferrin receptor, but also catalyze the conversion of H_2_O_2_ into oxygen. On this basis, Zhang et al. (2019b) developed a PEG functionalized liposome to load both holo-Lf and DOX for enhanced CMT and RT through relieving the hypoxic TME. As a result, 4T1-tumor average total O_2_ saturation improved from ∼4 to ∼35%, the hypoxia positive regions in tumors decreased from 60.2 to 17.3%, and HIF-1α positive areas reduced from 65.0 to 18.0%, respectively, due to the presence of holo-Lf. Utilizing two oxygen generators simultaneously could be better solve the tumor hypoxia problem. The iron oxide and CAT were conjugated to construct a biocompatible and biodegradable NPs (Cat-IONP) to reverse hypoxia-induced CMT resistance, which proved that the iron oxide can increase the enzymatic activity of free CAT by about 3 times in long-term enzymatic activity (Yen et al., 2019).

The success of MnO_2_, Pt and Fe in tumor hypoxia-reoxygenation has promoted researchers to find other nanozymes with similar catalytic properties. Cerium (Ce) oxide NP (nanoceria) is another category of high-efficiency oxygen generator, the oxidation state of cerium ion have a profound effect on H_2_O_2_ catalytic activity, cerium (IV) in oxidation state reveals a higher CAT-like activity than cerium (III) (Pirmohamed et al., 2010) as shown in reaction equation (8) and (9). Based on this mechanism, Yao et al. (2018) reported a synergetic one-for-all bio-photocatalyst based on lanthanide ion-doped mesoporous hollow cerium oxide upconversion NPs (Ce-UCNPs) with loaded DOX to realize tumor *in situ* oxygenation and potentiate the ROS-mediated cytotoxicity of the drug. *In vitro*, the generated oxygen reached 3.05 mg/L in 300 × 10^–6^ M H_2_O_2_ solution at the physiological pH environment (pH∼7.4) in 30 min, while the generation efficiency of oxygen decreased to 2.18 and 1.41 mg/L at the acidic pH∼5.5 and pH∼4.5, respectively. After i. v. injection of Ce-UCNPs in mice, the blood oxygen saturation in the tumor region increased from 6.9 to 19.2% in 100 min owing to their superior CAT-like activity. In addition, the doped lanthanide ions (Yb^3+^, Tm^3+^) can convert NIR light to UV emission to trigger the photocatalysis reaction catalyzed by cerium oxide matrix, effectively breaking oxygen and water into oxygen free radicals and hydroxyl free radicals. These reactive free radicals caused tumor cell death by apoptosis. To efficiently and stably break down H_2_O_2_ to oxygen in a weakly acidic TME, Jia et al. (2019) developed a mesoporous core-shell-structured NC with comprising of upconversion NPs core and a porous cerium oxide shell, and DOX was stored in the internal space. Clearly, the quantity of oxygen produced reaches 2.06 and 1.32 mg/L in H_2_O_2_ solution within 30 min at pH values of 5.5 and 4.5, respectively. After treatment, the cerium oxide-based NC can reach the tumor site via enhanced permeability and retention (EPR) effect to achieve *in situ* oxygen generation, thereby exhibiting an excellent tumor inhibition performance.

#### Others

Compared with nanozymes, some other O_2_ generating strategies that can efficiently alleviate tumor hypoxia but relatively few have been reported. For instance, metal oxides can be decomposed to produce oxygen under certain conditions. Chen Z. et al. (2018) constructed a multifunctional nanocomposite for treating hypoxia based on the oxygen release capability of copper oxide (CuO) triggered by microwave. As shown in equation (10), upon microwave treatment, the CuO in the cavities of NCs can be decomposed into cuprous oxide (Cu_2_O) and oxygen. *In vitro* study suggested that 2 mg/ml of the NCs dissolved in PBS at pH value is 5.5 can produce 7.28 mol/L of the dissolved oxygen. The *in vivo* result is consistent with *in vitro* study, which well indicates the oxygen production capacity of this CuO-based NCs. Similar to CuO, the gold trioxide (Au_2_O_3_) can self-decompose into oxygen under the light irradiation as equation (11), which can also be employed to relieve tumor hypoxia (Zhang et al., 2019a). More interestingly, recently it has been discovered that water can be utilized as an oxygen source to relieve tumor hypoxia. In a nice work from Zheng et al. (2016), the carbon dots were synthesized and deposited onto the C_3_N_4_ (CCN) to act as a water-splitting material. Under the red-light irradiation, the CCN could efficiently generate oxygen in water, as shown in equation (12). After treatment to 4T1 mice, the hypoxic TME was effectively alleviated and thus the expression of HIF-α and carbonic anhydrase 9 (CA9) were decreased, which was primarily attributed to the water splitting triggered oxygen generation capacity of CCN.

### Oxygen-Carrying Strategies

Oxygen-carrying NCs are mainly constructed from biosafe materials that have a high capacity to physically dissolve or chemically combine oxygen in *in vitro* of the oxygen-enriched environments and specific release oxygen in *in vivo* of the tumor hypoxic areas, so as to surmount hypoxia. Among these materials, the most commonly used to date are mainly PFCs and their derivatives, Hb, and MOFs.

#### Perfluorocarbons-Based Nanocarriers

PFCs, as an FDA approved oxygen carrier, have a strong ability to physically dissolve large amounts of oxygen through weak Van der Waals interactions and to deliver oxygen into hypoxic tumor tissues via passive diffusion. It has been confirmed that PFCs-based oxygen can rapidly and extensively relieve tumor hypoxia since oxygen dissolution in PFCs is dependent on the pO_2_. Spahn (1999) indicated that liquid PFCs can carry almost two-fold higher amounts of oxygen than whole blood owing to their excellent oxygen affinity. In addition, PFCs are chemically and biologically inert *in vivo* which the oxygen-carrying capacity is not affected by the pH, temperature, and blood circulation. Therefore, PFCs are extensively explored as oxygen-carries to attenuate tumor hypoxia. However, PFCs are highly immiscible in both lipophilic and hydrophilic solutions, which have to be stabilized by NCs to form nano-size formulations for clinical application. Considering the relatively long time required for various treatments to take effect, sustained hypoxia relief is desirable to maximize the efficacy. To achieve this goal, Zhou et al. (2019) ingeniously fabricated a PFC-loaded hollow Fe_3_O_4_ magnetic nanoplatform (PHMNP) capable of retaining in tumor cells for a long time through aggregation. Results show that oxygen could be released at a moderate rate from the PHMNPs over an extended period, thus the tumor hypoxia was effectively modulated. Of note, relieving tumor hypoxia can also be realized by inhibiting the platelet activity to promote RBCs distribution in tumors, since platelets have a role in the maintenance of abnormal tumor vessel barriers, which restricts RBCs to carry oxygen from blood to tumor tissues. On this basis, Wu et al. chose Perfluorotributylamine (PFTBA), a type of the PFC, to develop PFC@HSA NPs through the unfolding/self-assembly method. Their results suggested that the PFTBA@HAS NPs could inactivate platelets to induce the rearrangement of endothelial cells and tight junctions and eventually led to platelet removal through the reticuloendothelial system (RES). Hence, the oxygen-absorbed PFTBA@HSA NPs possess two-stage oxygen delivery, that is, oxygen evaporation from PFTBA as the first stage and elevated RBC infiltration as the second stage, which was demonstrated to be effective in reliving the hypoxic level (Zhou et al., 2018a; Zhou et al., 2018b). In addition to the PFTBA, some other PFC derivatives such as Perfluoropentane (PFP) (Chen S. et al., 2018) and Perfluorooctylbromide (PFOB) (Song et al., 2019), are also good oxygen carriers, which have been studied to relieve tumor hypoxia (Table 4).

#### Hemoglobin-Based Nanocarriers

Hb, as a natural oxygen carrier through chemical conjugation, can bind and penetrate tumor cell phospholipid membrane owing to hydrophobic force between them (Yang J. et al., 2018), thereby, it should be a competitive candidate for hypoxia reversal and subsequent therapies. However, some defects such as short circulation time, renal toxicity and cardiovascular complications severely restrict free Hb application. Besides, when the ferrous ion (Fe^2+^) in Hb is oxidized to three iron ions (Fe^3+^), the oxygen-carrying capacity will be deprived. Therefore, proper protection through incorporating Hb into the NCs to circumvent its shortcomings and ensure the intact conformation of binding sites, is essential for oxygen delivery. Liposomes can encapsulate Hb in its inner water phase for protecting the oxygen-carrying function, which can be employed to efficiently decrease the expression of HIF-α and VEGF in human colon carcinoma cells (Qu et al., 2017). Also, Hb can also be bounded to the surface of the liposome membrane by hydrophobic interaction, promoting the reversal of tumor hypoxia (Yang J. et al., 2018). Apart from liposomes, Hb molecules can also be formulated with the NP in the application of tumor oxygenation. Luo Z. et al. (2018) hybridized Hb and albumin NPs via disulfide reconfiguration and fabricated the tumor-targeted hybrid protein oxygen carriers (HPOCs) for precise tumor oxygenation. After i. v. injection, such Hb-based NPs can significantly modulate tumor hypoxia by donating bound oxygen deep in the tumor. Importantly, hypoxia relief inhibited the expressions of HIF-1α, multidrug resistance gene1 (MDR1), and P-gp; meanwhile, the abundant oxygen enhanced the ROS generation, which remarkably reduced hypoxia-induced CMT resistance and potentiated ROS-mediated cytotoxicity. Similar to this design, Hb, as an oxygen carrier, was connected with Ce6 to construct a 2-in-1 NP with Sorafenib (SOR) loaded to amplify the tumor-destroying effect (Xu et al., 2020). In their results, the *in vitro* oxygen release profile in Hb-Ce6 was significantly similar to that of the Hb group, and the Hb and Hb-Ce6 groups both showed significantly reduced HIF-1 expression, indicating that no impairment on the oxygen-carrying capacity occurred during the synthesis process of He-Ce6. As they expected, such a Hb-based NP can significantly boost the generation of ROS and enhance ferroptosis by improving tumor oxygen level.

In addition, RBCs are also an attractive choice for the reoxygenation of tumor tissue due to their inherent biocompatibility, long blood circulation ability, low immunogenicity, and easy availability. Although the size of RBCs is about 5∼7 μm, which technically does not meet the definition of ‘NC’, it is still necessary to discuss the applications of RBCs in hypoxia relief owing to their high oxygen-carrying capacity. Tang et al. (2016) introduced a new technology called RBC-facilitated PDT, or RBC-PDT, to potentially address the tumor hypoxia issue. Briefly, ZnF16Pc acts as a photosensitizer that was encapsulated into ferritin nanocages (P-FRT) and then the nanocages were conjugated to the surface of RBCs through biotin-avidin coupling. They hypothesized that O_2_ liberated from RBCs would not completely relieve the tumor hypoxia, but the O_2_-rich region close to the surface of RBCs could continue to induce production of ^1^O_2_. After treatment to U87MG subcutaneous tumor models, as expected, they found that RBCs can provide O_2_ to enable sustained ^1^O_2_ production even when P-FRT-RBCs were under low oxygen conditions. In another highlight, a remote activable smart drug delivery system for site-specific tumor hypoxia was designed by modifying RBCs with orthogonal excitation-emission upconversion NPs functionalized with an ultrasensitive hypoxia probe (HP) and the PDT photosensitizer (Rose Bengal). Under NIR excitation, the inactive HP can be transformed into an active state specifically to trigger the O_2_ release from oxygenated Hb in a hypoxic TME. Moreover, such an RBC oxygen carrier can also overcome a series of complex biological barriers such as reduced RES uptake, evaded mononuclear phagocyte system, and transported across the inflamed endothelium (Wang et al., 2017). However, the size of RBC is too large to penetrate the deep inside tumors, let alone the hypoxic tumor cells. Hence, more attention is now being focused on the development of nanoscale RBC-mimicking systems.

#### Metal-Organic Frameworks-Based Oxygen-Carrying Nanocarriers

MOFs, a type of crystalline hybrid materials synthesized by coordinating metal ions with multitopic organic linkers, are also named porous coordination polymers or porous coordination networks. Due to high porous structures, ultrahigh surface area, uniform pore sizes and potential biomedical applications, MOFs used as an oxygen carrier are also emerging as fascinating NCs for tumor hypoxia reversion. There are four common strategies for loading therapeutic cargoes into MOFs including noncovalent encapsulation, conjugation to the linkers, use of therapeutics as linkers, and attachment to the secondary building units (Lu et al., 2018). For example, Gao et al. (2018) reported a biomimetic oxygen-evolving PDT NC featured with long circulating to eliminate hypoxic tumors. That is, Zirconium (IV)-based MOF (UiO-66) was used as a vehicle for oxygen storing, then conjugated with ICG by coordination reaction, and further coated with RBC membranes. Upon laser irradiation, the initial ^1^O_2_ produced by ICG would destroy RBC membranes, and facilitated the burst release of oxygen from UiO-66. Owing to the advantages of long circulation and oxygen self-sufficient, such a MOF-based NC showed excellent tumor accumulation and oxygenation leading to markedly decreased hypoxia. Taking advantage of the oxygen-carrying capacity of Zeolitic imidazolate framework-90 (ZIF-90, a type of MOF), Xie et al. (2018) fabricated an oxygen-loaded pH-responsive multifunctional NCs for hypoxia modulation. Briefly, a core of upconversion NPs was first coated with mSiO_2_ and loaded with Rose Bengal, ZIF-90 was coated outside of mSiO2, DOX and tumor-targeted molecule PEG-FA were covalently conjugated on the surface of the system. Under acidic TME, ZIF-90 can quickly be decomposed to release oxygen, thus, Rose Bengal as a photosensitizer produced large amounts of ROS due to the hypoxia alleviation.

### Increasing Intratumoral Blood Flow

Apart from oxygen-generation and oxygen-carrying, the increasing local temperature of a tumor by mild hyperthermia to improve intratumoral blood flow can also alleviate the hypoxia issue. Accumulating evidence has shown that the photothermal effect of near-infrared absorbing nano-agents could generate hyperthermia through converting photonic energy into heat. Therefore, mild photothermal therapy (PTT) with various nanomaterials would be favourable for tumor hypoxia relief. Based on this inspiration, a partial cation exchange method was employed by Liu research group to fabricate a versatile MnSe@Bi_2_Se_3_ core-shell nanostructure. Both *in vivo* fluorescence imaging utilizing a hypoxia-specific probe and *ex vivo* immunofluorescence staining suggested that mild PTT by low-power laser irradiation after injection with MnSe@Bi_2_Se_3_ NPs significantly reduced tumor hypoxia *via* improved intratumoral blood flow (Song et al., 2015). Li group introduced a versatile nanomaterial based on MoS_2_ quantum dot@polyaniline (MoS_2_@PANI) inorganic-organic nanohybrids, which also exhibit a good potential to enhance tumor oxygen level by increasing intratumoral blood flow (Wang J. et al., 2016). Besides hyperthermia, anti-angiogenic drugs to normalize tumor vasculature can also increase tumor blood flow and subsequently achieve effective oxygen perfusions, such as gemcitabine (Cham et al., 2010) and cyclophosphamide (Emmenegger et al., 2006).

### Decreasing Intratumoral Oxygen Consumption

As an alternative to improving the oxygen supply to the tumor, oxygenation could also be increased by decreasing intratumoral oxygen consumption. Metformin (Met) exerts a potent cellular respiration inhibitory effect by directly suppressing the activity of complex I in the mitochondrial electron transport chain. It is mainly used in the clinical treatment of type II diabetes. However, Zannella et al. (2013) demonstrated that Met can effectively improve tumor oxygenation by reprogramming cell metabolism. Liu et al. reported an innovative use of Met to achieve tumor hypoxia relief. In their design, hydrophilic Met and modified hydrophobic Ce6 were co-encapsulated in the internal water phase and bilayer membrane of nanoliposomes, respectively. Due to EPR effect, the i. v. injection of such Met-based liposomes greatly improves tumor oxygenation in 4T1, CT26, and SCC-7 tumor models, which the positive hypoxia area decreased from 56 to 15%, from 81 to 32%, and from 73 to 33%, respectively (Song et al., 2017). In a separate study, Met and W_18_O_49_ NPs were co-loaded into platelet membranes to relieve the hypoxic level via reducing oxygen consumption (PM-W18O49-Met NPs). As anticipated, the low oxygen consumption rate of tumor cells caused by accumulated Met in the tumor site could overturn hypoxia (Zuo et al., 2018).

## Regulating Properties of Oxygen-Based NCs for Increasing Their Targeting Ability to Hypoxic Cells

**FIGURE 5:**
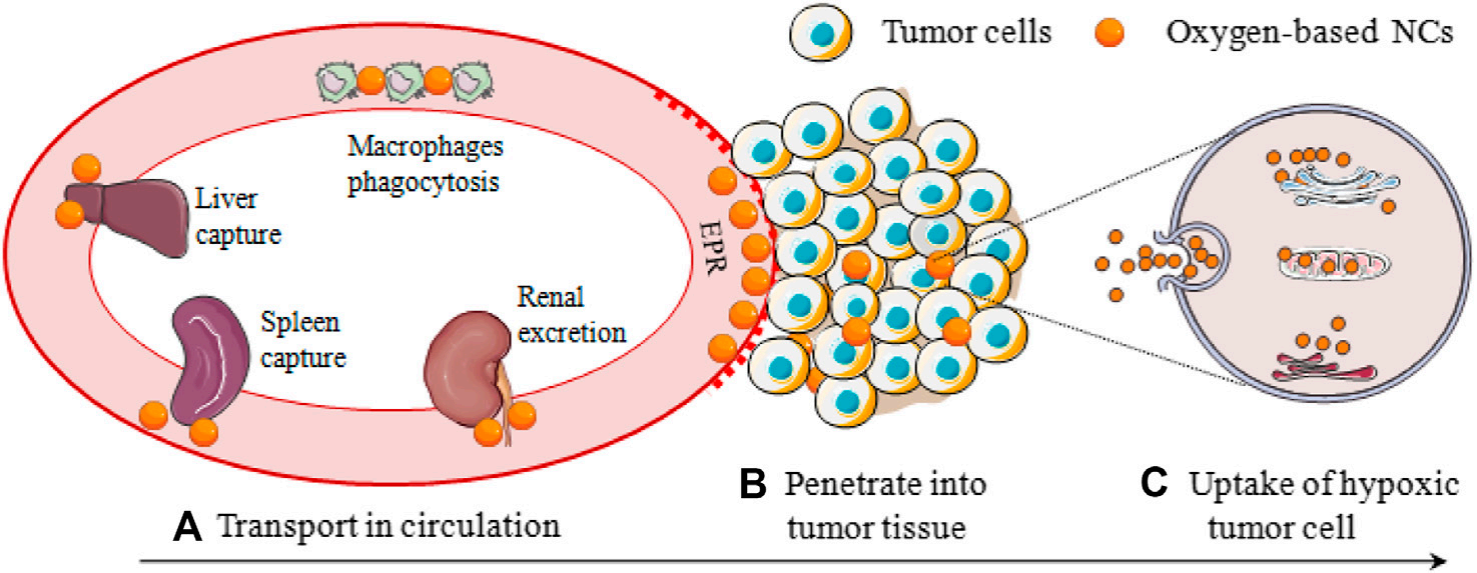
Hypoxic tumor cells oxygen-based NCs delivery process, involving **(A)** Transport in circulation; **(B)** Penetrate into tumor tissue; and **(C)** Uptake of hypoxic tumor cell.

However, these well-designed oxygen-based NCs are difficult to accurately target the hypoxic tumor cells, which can be attributed to 1) NCs in the blood circulation need to overcome various physiological barriers such as RES phagocytosis, liver and spleen capture, and kidney filtration before accumulating the tumor sites; 2) tumor tissues featured dense stroma, abnormal angiogenesis, and elevated interstitial pressure, which severely restricted the penetration of the oxygen-based NCs to deep areas where the hypoxic cells are located; 3) NCs are difficult to adhere or bind to hypoxic tumor cells, thus the cellular uptake is always inefficient (Figure 5). Besides, the EPR effect of leaky tumor vasculature is often over-represented, more than 95% of NPs are reported to be accumulated in normal organs (Bae and Park, 2011). He et al. (2010) demonstrated that physicochemical and surface properties of NPs and liposomes were more important parameters than their composition. It is reported that the size of oxygen-based NCs profoundly affects their blood circulation and tumor penetration and the targeting ligand determines the process of uptake by tumor cells. In this section, the size and targeting ligand of oxygen-based NCs was emphatically introduced with special attention to the latest fabrications and nano-technologies, and the focus is on the fate of the NCs *in vivo* after i. v. administration.

### Size-Switchable Oxygen-Based NCs

The size of NCs can be controlled by adjusting temperature, stirring rate, pH, reaction time, major reactant, etc. in the synthesis process. Size is a key factor in determining biological and pharmacokinetic (PK) behaviour in the body. NCs with relatively large sizes (∼50–100 nm) have been reported to reduce capture by the liver and spleen, increase RES avoidance, decrease renal excretion, and reduce the formation of protein corona to weaken phagocytosis by phagocytes (Gabizon and Papahadjopoulos, 1988; Wang et al., 2015). In return, prolonged blood circulation time and efficient tumor accumulation through the EPR effect can be achieved, but are also limited by poor permeability and distribution in the dense extracellular matrix and tumor-associated fibroblasts (Li et al., 2016). On the contrary, NCs with ultrasmall sizes below 10 nm typically show greater tumor penetration because of their reduced diffusional hindrance (Zhang et al., 2016), which is also supported by the Stokes-Einstein equation. Besides, small particle size generally suffers from shorter circulating half-life time and inferior tumor accumulation. Such a dilemma has driven researchers to develop size-switchable oxygen-based NCs that can maintain a large initial size (∼100 nm) in the blood circulation and dissociate into small particles (∼10 nm) to achieve deep penetration after reaching the tumor site.

According to existing literature, the size-switchable oxygen-based NCs can be realized by responding to acidic TME. In a design from Chen J. et al. (2017), BSA-Au nano-complexes were first synthesized and as a template coated with manganese chloride (MnCl_2_) molecules to form MnO_2_ NPs in alkaline conditions through biomineralization. The particle size of the obtained BSA-Au-MnO_2_ NP was about 60 nm at a neutral pH of 7.4, but the size would be gradually decreased at an acidic pH of 6.5. Especially in the presence of H_2_O_2_, almost all BSA-Au-MnO_2_ NPs under pH 6.5 decreased to 10 nm in size within 2 h. *In vivo* biodistribution data confirmed a higher tumor uptake of BSA-Au-MnO_2_, which should be attributed to the more effective EPR effect of tumors for NPs with suitable sizes. In intratumoral diffusion study, the Au nanoclusters fluorescence signals of tumor slices from mice injected with BSA-Au-MnO_2_ were located far from blood vessels, suggesting that BSA-Au-MnO_2_ in the presence of acidic TME and endogenous H_2_O_2_ would be degraded into small NPs of less than 10 nm to penetrate deeply. Thanks to the switchable particle size feature, the tumor hypoxia positive area decreased from 20% to about 2%. Tian et al. (2017) developed radionuclide ^131^I labelled HSA-bound MnO_2_ NPs (^131^I-HSA-MnO_2_) that can responsive to acidic pH/H_2_O_2_. In blood at pH of 7.4, the size of as-made NPs was about 50 nm, which exhibited longer blood circulation time and greater tumor homing ability. The biodistribution in normal tissues were remarkably decreased. These NPs can be gradually degraded and decomposed into individual ^131^I-HSA below 10 nm in an acidic TME, and thus the intratumoral diffusion of HSA-NC could be dramatically enhanced. In a similar undertaking from Chen et al. (2016), HSA was first conjugated with either photosensitizer or pro-chemodrug to yield individual albumin-drug complexes, and then dispersed in MnCl_2_ solution under vigorous stirring, so that the Mn^2+^ can be anchored to the active groups of HSA to form HSA-Mn complexes. Meanwhile, using NaOH to build an alkaline environment to promote the oxidation of MnCl_2_ to MnO_2_, so that the generated MnO_2_-based NC with a particle size of about 50 nm can respond to the acidic TME and H_2_O_2_ to gradually degrade into individual therapeutic albumin complexes with less than 10 nm sizes. Upon systemic injection into mice, the blood circulation half-life of the size-switchable NC was 21.239 ± 4.5 h, while that of the control group was 4.69 ± 0.78 h. The deep penetration barrier of tumor-associated fibroblasts and condensed extracellular matrix was also well addressed.

Besides, size-switchable oxygen-based NCs can also be obtained by responding to hypoxic TME. As an example, a unique type of hypoxia-responsive HSA-based NC was prepared by Yang et al. (2019) via cross-linking the hypoxia-sensitive azobenzene group between Ce6-conjugated HSA and chemodrug-conjugated HSA. The as-made NC can be stable under normal pO_2_ with a diameter of 100–150 nm. Under exposure to hypoxic TME, the azobenzene group in NCs will be cleaved by reductase and quickly decomposed into ultrasmall therapeutic NPs with a size of less than 10 nm. In another attempt, Chen Q. et al. (2017) fabricated a size-changeable HSA-Ce6-CAT-PTX NPs via a simple one-step method. The initial particle size of the NP was about 100 nm. Once enter the tumor site, the concentration of these NPs would be gradually decreased to trigger their dissociation into smaller CAT-based NPs, thereby enabling enhanced penetration inside the tumor and effectively decomposing H_2_O_2_ into oxygen. *In vivo* study demonstrated that the expression of HIF-1α remarkably reduced and the hypoxia positive area decreased from 32 to 7%.

### Oxygen-Based Nanocarriers With Specific Targeting

The targeting effect of the oxygen-based NCs is to increase the accumulation of particles at tumor sites as much as possible. The NCs targeting can be divided into passive and active targeting. Passive targeting of NCs is based on the physicochemical and surface properties and on a prolonged blood circulation, which leads to their accumulation in tumors via the EPR effect caused by vascular leakage and lymphatic defects/lacks of tumors (Bae and Park, 2011). However, controversy still surrounds the concept of EPR, and the existence and implication in humans remain unresolved (Bae and Park, 2011). In contrast, active targeting is achieved by modified NCs that serve as guided missiles (that is, ligands grafting) to deliver the loadings to tumor sites. Active targeting exhibits a better tumor accumulation and is currently pursued in fields of tumor hypoxia relief. HA, a biodegradable and biocompatible polymer composed of alternating units of two sugar monomers, is known to target over-expressed CD44 receptors in many tumors such as breast carcinoma and lung carcinoma. Also, HA is highly amenable to modify NCs for tumor targeting due to the presence of reactive functional groups such as hydroxyl, carboxyl, acetamide groups, etc. Inspired by this, Gao et al. (2017) developed HA-based MnO_2_ NCs to enhance tumor targeting. Results from their study revealed that the tumor/muscle ratio at 6 h post-injection was 4.03 ± 0.36 for fluorescent imaging and 2.93 ± 0.13 for photoacoustic imaging, indicating that such a HA-based NCs can efficiently accumulate at tumor sites. This remarkable tumor targeting ability of HA modification was also well utilized by Phua et al. (2019) and Song M. et al. (2016) in engineering CAT-encapsulated HA-based NPs and HA-coated MnO_2_ NPs, respectively. The over-expression of folate (FA) receptor on tumor cells including breast, lungs, kidneys, ovaries, colon, brain, etc. has also been exploited for delivery of FA-modified oxygen-based NCs to tumors, such as FA-modified CAT-based liposomes for breast tumor hypoxia alleviation (Shi et al., 2020). For more efficient receptor-mediated oxygen-based NCs tumor accumulation, FA is often conjugated with PEG to modify the NCs, such as FA-PEG MnO_2_-based NCs (Liu et al., 2019) and FA-PEG MOF-based NCs (Xie et al., 2018). Apart from HA and FA, some other ligands are also chosen to bind to a receptor up-expressed by tumor cells or tumor vasculature and not expressed by normal cells for active targeting (Zhong et al., 2014).

Noteworthily, specific targeting subcellular organelles are also a practicable strategy to overcome hypoxia-induced resistance (Chen et al., 2019). For example, Cheng et al. (2019) applied a chimeric peptide and designed a self-delivery NP for mitochondria and plasma membrane dual-targeted, which facilitated the production of intracellular ROS during PDT. Importantly, plasma membrane-targeted disrupted the cell membrane and mitochondria-targeted decreased mitochondrial membrane, which significantly induced cell apoptosis. Both *in vitro* and *in vivo* results indicated that this combinatorial PDT had a maximized therapeutic effect and a minimized side effect owing to the dual-targeted function. In another research, Li W. et al. (2019) employed endoplasmic reticulum (ER)-targeting pardaxin peptides and fabricated a Hb-based liposome to alleviate tumor hypoxia. Under NIR light irradiation, such an ER-targeting NC can induce intense ER stress and calreticulin exposure on the cell surface and significantly activate a series of immunological responses, which greatly enhanced anti-tumor efficacy of PDT and PTT. Zinc (II)-phthalocyanine, as a photosensitizer, has a capable of targeting golgi apparatus, was utilized by Acedo et al. (2014) to boost PDT. After i. v. injection into tumor-bearing mice, such a photosensitizer was preferentially accumulated in the tumor region, and induced the activation of golgi caspase-2 in A-549 cells and triggered apoptosis. Thus, golgi-targeted PDT could also effectively destroy the hypoxic tumor cells.

### Other Physicochemical and Surface Properties

Other physicochemical properties (e.g., morphology, elasticity) and surface properties (e.g., charge, hydrophobicity) of oxygen-based NCs also profoundly affect tumor hypoxia relief. However, most previous studies have designed oxygen-based NCs without investigating the influence of other physicochemical and surface properties on their various *in vivo* processes. To this end, the appropriate morphology, elasticity, surface charge, and hydrophobicity of oxygen-based NCs to extend blood circulation time and increase tumor penetrability were introduced, which were based on the field of NCs.

The morphology of NCs is mostly designed to be spherical, but the unique properties of non-spherical NCs may provide new ideas in the design of NCs for tumor hypoxia modulation. Recent data has shown that non-spherical NCs such as rod, ellipse, cylinder, ring, star, and other irregular morphologies had a distinctive circulation time owing to different behaviour in aligning with the blood flow (Arnida et al., 2011), and exhibited an enhanced tumor targeting and accumulation, which should be attributed to better margination, adhesion, and extravasation of aspheric NCs, and less return of NCs out of the TME (Christian et al., 2009). Elasticity refers to the ability of NCs to be deformed, which is considered to be an important factor influencing the fate of NCs *in vivo*. Research has found that softer NCs exhibit significantly reduced cellular uptake in macrophages, enhanced circulation and subsequently enhanced tissue targeting when compared to the harder NCs. The softer particles can deform and resist movement out of the leaky vasculature, which can also be utilized for the tumor-specific targeting applications (Key et al., 2015). In general, surface charge could influence the adsorption of opsonins, thus leading to the recognition, phagocytosis and elimination of NCs by macrophages, which in turn affects their various *in vivo* processes. As reported in the literature, high surface charges, either positive or negative, speed up blood clearance and enhance RES capture while the near-neutral charges contribute to improved RES avoidance and extended blood circulation (Xiao et al., 2011). NCs surface charge also plays a critical role in tumor accumulation. But differently, NCs with a higher positive charge density tended to be accumulated in tumor more efficiently. Besides, electropositive NCs have been suggested to lead to better tumor tissue penetration and tumor cell uptake via electrostatic attractions (Wang H. X. et al., 2016). Therefore, NCs should be slightly negatively charged or uncharged during blood circulation, and once located and accumulated in the tumor tissue, the surface of NCs needs to be positively charged. Yuan et al. (2012) fabricated a charge-switchable NP based on zwitterionic polymer by introducing a tumor extracellular acidity sensitive group as the anionic part of the zwitterionic polymer. The surface charge of the NPs is near-neutral in the blood and reduces protein adsorption, thus prolonging the blood circulation time. After accumulating in the acidic TME via EPR effect, such NPs switched to be positively charged by exfoliating the anionic group, leading to a remarkable enhancement in tumor penetration. NCs with a hydrophobic surface will absorb plasma proteins after i. v. injection and the more hydrophobic the surface, the more proteins will be adsorbed, thus undergo faster blood clearance and capture by the RES. Accumulating evidence has shown that conjugating the hydrophilic materials to change the surface hydrophobicity of NCs is one of the most effective ways to increase the RES avoidance and extend the cycle time (Li Y. et al., 2019). Among these hydrophilic materials, PEG is the most widely used. However, a higher surface density of hydrophilic nanomaterials will restrain tumor penetration, cellular uptake, and intracellular trafficking of NCs. Xu M. et al. (2019) developed PEG-detachable pH-responsive polymeric micelles. After accumulating at the tumor site, the acidic TME cleaved the unstable linker between the PEG segment and the main chain, resulting in the detachment of the PEG shell and protonating the NCs, thus facilitating cellular uptake of the NCs by tumor cells, along with the escape from endo-lysosome due to the “proton-sponge” effect. In sum, hydrophilic materials such as PEG can reduce the RES effect of NCs, whereas hydrophobic NCs surface can increase penetration and uptake efficacy. Therefore, PEGylation in blood and PEG-detachment in tumor sites should be a good choice.

## Oxygen-Based Nanocarriers to Enhance Tumor Therapies

**FIGURE 6:**
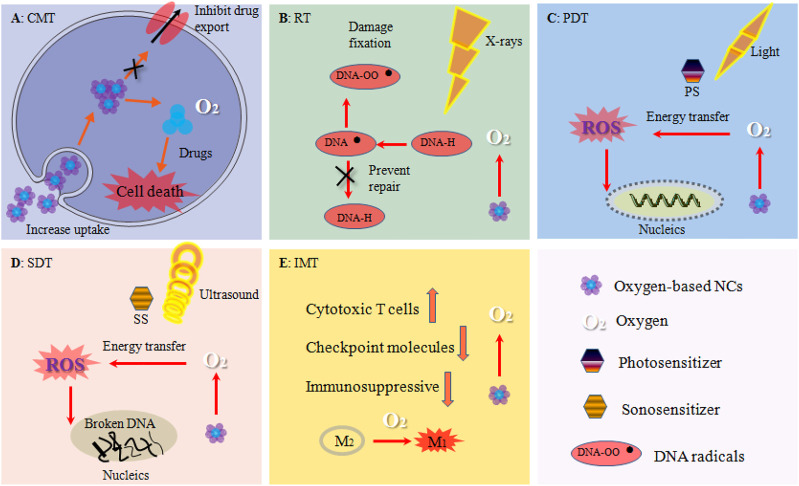
Oxygen-based NCs boosted tumor therapies. **(A)** CMT: alleviated hypoxia decreased the expression of drug efflux pumps such as P-gp and increased drugs uptake by tumor cells. **(B)** RT: oxygen stabilized DNA damages and prevented DNA self-repair by cells. **(C)** PDT: oxygen enhanced the generation of ROS such as ^1^O_2_. **(D)** SDT: elevated ROS level enhanced DNA broken. **(E)** IMT: oxygen increased the number and activity of cytotoxic T cell, down-regulated the expression of important immune checkpoint molecules such as PD-L1, changed the immunosuppressive TME, tilted the polarization of TAM from the M2 phenotype to the tumor-inhibiting M1 phenotype.

Oxygen-based NCs have significantly boosted various tumor treatment strategies including CMT, PDT, SDT, RT, IMT, and their combinations (Figure 6). The detailed *in vivo* tumor growth inhibition (TGI) and the mechanisms were shown in Table 3 and Table 4. Some representative studies and discussion are shown as following.

### Monotherapies

#### Chemotherapy

Oxygen-based NCs can remarkably reduce hypoxia-induced CMT resistance and increase ROS-mediated cytotoxicity. Hb-based nanoliposome with loaded DOX showed efficiency inducing the reversal of tumor hypoxia, increasing the uptake of the DOX into tumor cells, and inducing a significantly increased toxicity of the drug against tumor cells (Yang J. et al., 2018). Zhou et al. (2019) designed a PFC and etoposide (EP) loaded porous hollow Fe_3_O_4_-based NC capable of relieving tumor hypoxia. Results from their study revealed that hypoxia could be alleviated at a moderate rate and the intracellular EP level was significantly elevated, thus effectively decreasing the hypoxia-induced CMT resistance. Similarly, PFOB nanoemulsion has proven to have great potential to overcome hypoxia-induced cisplatin resistance (Song et al., 2019). PFTBA-based HSA NP capable of enhancing oxygen carrier RBC distribution in tumor site was originally engineered by Zhou et al. (2018b), which significantly amplified CMT efficacy with *in vivo* TGI of more than 80% by improving tumor oxygen concentration.

#### Radiotherapy

Attenuating tumor hypoxia by oxygen-based NCs has been proven to be effective in enhancing RT efficacy (Rey et al., 2017). Indeed, oxygen molecules can stabilize DNA damages caused by ionizing radiation to prevent DNA self-repair by cells, thus enhancing cell killing during RT. For instance, Liu group designed a novel type of bio-nanoreactors by encapsulating CAT within TaOx nanoshells via a simple and mild one-step method. After decomposing H_2_O_2_ into oxygen, the hypoxia-associated RT resistance was greatly relieved, which exhibited more than 95% *in vivo* TGI (Song G. et al., 2016). Prasad et al. (2014) fabricated multifunctional and colloidally stable bioinorganic NPs composed of polyelectrolyte albumin complex and MnO_2_ to enhance RT response. They have shown that the DNA double-strand breaks and tumor cell death greatly increased. Chen J. et al. (2017) engineered a size-changeable BSA-Au-MnO_2_ composite NP to increase tumor permeability of oxygen-based NCs. As a result, highly effective RT (*in vivo* TGI >90%) of tumors is realized with those NPs in a mouse tumor model. They also developed a size-switchable radionuclide ^131^I labelled HSA-bound MnO_2_ NPs, which also showed great RT efficacy in tumor treatment upon systemic administration (Tian et al., 2017). Pt, as a high-Z radio-sensitizing element, not only can catalyze H_2_O_2_ into oxygen, but also can enhance radiation dose to tumor cells via effectively emitting electron radiation after interaction with X-rays. Taking this advantage, Li Y. et al. (2019) proposed porous Pt NPs as a new oxygen-based NC for RT enhancement. As they expected, the RT-induced DNA damages, cell cycle arrest, and ROS stress were significantly increased by porous Pt NPs and greatly amplified the efficacy of RT. PFCs with dual ability to re-model tumor hypoxia were also extensively explored to increase the therapeutic outcome of RT. As an example, Zhou et al., 2018a prepared a simple but effective PFTBA-HSA NP that can reverse hypoxia-induced RT resistance in two stages. That is, PFTBA releases the physically dissolved oxygen and promotes the RBCs infiltration in tumor sites to release oxygen. As anticipated, the tumor growth rate in response to RT of hypoxic breast cancer and colon cancer decreased from 40 to 14% and from 30 to 15%, respectively.

#### Photodynamic Therapy

With the help of oxygen-based NCs, tumor hypoxic TME can be effectively reversed or relieved, resulting in a significant increase in the production of ^1^O_2_ under local irradiation, thus greatly boosting PDT efficacy (Sun et al., 2020). To improve the efficacy of PDT limited by hypoxia, a cell-specific, H_2_O_2_-activatable, and O_2_-evolving PDT NP composed of MB and CAT in the aqueous core, BHQ-3 in the polymeric shell, and functionalized with a targeting ligand, was fabricated by Chen and his colleagues. Under irradiation, the continuously generated ^1^O_2_ in PDT process efficiently destroys tumor cells, and the *in vivo* TGI was as high as 100% (Chen et al., 2015). Gao et al. (2017) developed an oxygen-generating NC by encapsulating a MnO_2_ NP in an ICG modified HA NP to overcome tumor hypoxia. After laser irradiation, about 100% *in vivo* TGI was observed in such an oxygen-generating NC group, which should be attributed to the elevated level of oxygen. Complete *in vivo* tumor growth inhibition of MnO_2_-based NCs was also found by Liu et al. (2019). In addition to CAT and MnO_2_ catalysts, Pt and Pd nanoplates modified with PEG and conjugated with Ce6, which can modulate hypoxic TME to enhance the PDT effect, was successfully fabricated by Zheng group. Results from their study suggested that the tumors could be eliminated at the 6th day post-injection (Wei et al., 2018). For oxygen carrying strategies, Gao et al. (2018) employed Zirconium (IV)-based MOF as a vehicle for oxygen storing and conjugated with ICG and further coated with RBC membranes to ablate tumor growth. Upon 808 nm laser irradiation, the initial ^1^O_2_ produced by ICG decomposed RBC membranes to cause the burst release of oxygen from MOF, greatly enhanced ^1^O_2_ generation, thus remarkably improved the PDT effect. On the 27th day of i. v. administration, the tumor was completely eliminated.

#### Sonodynamic Therapy

SDT efficacy could also be augmented by oxygen-based NCs. Zhu P. et al. (2018) constructed a versatile nanosonosensitizer by the integration of MnO_2_ with hollow mesoporous organsilica NPs, and conjugated with sonosensitizer and targeting peptide. After injection to mice bearing U87 tumor xenografts, the inorganic MnO_2_ can effectively convert the tumor over-expressed H_2_O_2_ into oxygen and thus modulated tumor hypoxia, which has been demonstrated to facilitate SDT-induced ^1^O_2_ production and remarkable enhance SDT efficacy subsequently. At the end of the whole therapeutic evaluation, the *in vivo* TGI of the treatment group was as high as 96%, much higher than that of all the control groups. In another study, Zhang N. et al. (2020) developed a pH-responsive drug-loaded liposome to reverse hypoxia-induced RT resistance by reducing oxygen consumption. Results revealed that Met molecules released from liposomes can effectively inhibit the mitochondrial respiratory chain. Under ultrasound irradiation, such a liposome can greatly destroy breast tumors by enhancing the generation of cytotoxic ^1^O_2_, exhibiting an excellent SDT performance.

### Enhanced Chemotherapy by Synergistic Strategies

CMT plays a vital role both in treating tumors and easing tumor symptoms. However, mono CMT always cannot fully meet the current requirements of tumor treatment due to chemotherapeutics inferior physicochemical property and narrow therapeutic windows, and the complexity of biological processes involved in the pathogenesis of tumors. Therefore, taking advantage of the synergistic strategy that combines other therapies such as PDT, RT, IMT with CMT could achieve the effect of 1 + 1>2 and thus obtain a better tumor cell killing effect.

#### Chemotherapy-Photodynamic Therapy

Hypoxia regulates tumor gene/protein expressions involved in CMT resistance. To overcome this adversity, Luo Z. et al. (2018) fabricated well-defined hybrid protein oxygen carriers (Hb-HSA) with loading of DOX and Ce6 via disulfide reconfiguration. Results demonstrated that the expression of HIF-α, MDR-1, and P-gp significantly down-regulated, resulting in the minimized cellular efflux of chemodrug. Moreover, alleviated hypoxia also upgraded ROS generation during the PDT process. As a result, only a single-dose treatment of the Hb-HSA-based CMT-PDT improved the tumor inhibition rate to about 90%, while the control group less than 50%. With the same purpose, a versatile pH-responsive oxygen-based NC with loaded MnO_2_, g-C_3_N_4_ as visible-light photocatalyst and DOX was developed by Zhang W. et al. (2018). When *in vitro* treatment under light irradiation, the cell viability of 4T1 cells reduces to 4.5 ± 0.21% and exhibit significant apoptosis of 94.38%. *In vivo* results also indicated that 4T1 tumors can be completely eliminated by the designed NC under light irradiation. Beyond this, oxygen-based NCs can also solve the poor drug cellular uptake induced by hypoxia. As an example, a hollow-structured biophotocatalyst NP with coated mesoporous cerium oxide and loaded DOX was developed by Jia et al. (2019) to reverse CMT-PDT resistance. After 0.5 h of incubation of the NPs and HeLa cells, there was a small proportion of NPs that were internalized. With an increase in incubation time, more NPs were assimilated by tumor cells. Also, the CLSM images indicated that the as-prepared NPs were internalized in the cell instead of just being adhered to the membrane surface. The *in vivo* TGI was as high as 90%, which should benefit from the increase in drug uptake and the synergistic effect between CMT and PDT.

#### Chemotherapy-Radiotherapy

The combination of CMT and RT has been widely studied and applied in clinics for the improvement of tumor therapy. Zhang R. et al. (2017) reported CAT-loaded cisplatin-prodrug-constructed liposomes to relieve tumor hypoxia. Both *in vitro* and *in vivo* results clearly indicated that the obtained CAT-based NCs could trigger decomposition of H_2_O_2_ produced by tumor cells into oxygen. As expected, treatment of such a liposome induces the highest level of DNA damage in tumor cells and offers the most effective inhibition effect on tumor growth (85% *in vivo* TGI) after X-ray radiation compared to the control groups. Lactoferrin is a natural protein that exhibits multiple anti-tumor effects such as induced cell membrane disruption, immunoregulation, cell arrest, and apoptosis (Zhang Y. et al., 2014). Taking this advantage, Zhang et al. (2019b) designed PEG-modified liposome as NCs to load both holo-lactoferrin and DOX for combined CMT-RT of tumor. They found that holo-lactoferrin released from NCs could catalyze the conversion of H_2_O_2_ to oxygen. After one-time treatment plus X-ray radiation, the tumor volume of mice treated with the as-made liposome was significantly inhibited as compared to other groups. On the 26th day post-injection, the tumors were completely eliminated. In another intriguing study, a MnO_2_ functioned albumin-bound paclitaxel NP was fabricated by Meng et al. (2018) to remove the limitation of hypoxia on the efficacy of CMT-RT for colorectal cancer. Upon X-ray radiation, such MnO_2_-based NCs exhibited the most remarkable delay in tumor growth with a 96.57% inhibition in all treatments.

#### Chemotherapy-Immunotherapy

It is reported that CMT could strengthen the effects of IMT, which in reverse reduced CMT resistance (Lu et al., 2017). However, a hypoxic TME could frustrate IMT clinical efficacy through various mechanisms, one of which is up-regulating the expression of PD-L1, which results in the exhaustion of CD8^+^ T cells. To this end, a multifunctional biomimetic core-shell NP was developed by Zou et al. (2018) to enhance the therapeutic efficacy of CMT-IMT. In more detail, CAT and DOX loaded in the ZIF-8 was used as the core to generate oxygen and reserve chemodrug, while the murine melanoma cell membrane was used as the shell to provide tumor targeting ability and elicited immune response. After treatment with such a design and αPD-1, the tumor growth was almost completely inhibited, and the number of CD8^+^ T cells recruited to tumor was 35.5%, while all numbers in the control group were ∼20%. The expression of HIF-1α and PD-L1 down-regulated, resulting in enhanced CMT and immune response and inhibited the PD-1/PD-L1 axis, which significantly prolonged tumor recurrence time and inhibited tumor metastasis. TAMs located in the hypoxic area of tumors cooperate with tumor cells to promote proliferation and therapy resistance. To modulate CMT-IMT resistance, HA-coated, mannanconjugated MnO_2_ NP was constructed by Song M. et al. (2016) for targeting the TAMs. Combination treatment of such MnO_2_-based NP and DOX remarkably increased apparent diffusion coefficient values of breast tumor, inhibited tumor growth and tumor cell proliferation. More importantly, after successfully lessening hypoxia, the polarization of TAM tilts from the M2 phenotype to the tumor-inhibiting M1 phenotype.

### Other Combined Therapies

#### Photodynamic Therapy-Radiotherapy

The oxygen-dependent-featured PDT and RT are two representative non-invasive therapies that can efficiently induce tumor cell death by generating a great deal of ROS. Fan et al. (2015) engineered intelligent MnO_2_ nanosheets anchored with UCSMs to overcome hypoxia. Upon co-irradiation of NIR and X-ray, the treatment of UCSMs plus RT and NIR resulted in the lowest cell viability *in vitro* and the highest level of apoptosis/necrosis *in vivo*, which should be attributed to the synergetic effect of PDT-/RT to achieve higher anti-tumor efficacy.

#### Photodynamic Therapy-Immunotherapy

Liang et al. (2018) fabricated the core-shell Au nanocage@MnO_2_ NPs as TME responsive oxygen generators and NIR-triggered ROS producers for oxygen-boosted PDT-IMT against metastatic triple-negative breast cancer. In this study, MnO_2_ shell degrades in acidic TME pH/H_2_O_2_ conditions and releases sufficient oxygen to enhance the PDT effect of the NPs under laser irradiation. Meanwhile, alleviated hypoxia remarkably evoked systematic anti-tumor immune responses through increased recruitment of T cells in tumors, elicited immunogenic cell death, and altered the immunosuppressive TME. Experimental evidence from *in vivo* studies demonstrated that such NPs with laser irradiation not only completely ablated the primary tumor but also greatly inhibited the tumor metastasis and recurrence. Based on the Fenton reaction, Lan et al. (2018) fabricated an oxygen-based NC consisting of a nanoscale MOF and TBP to explore the synergistic combination of PDT and immune checkpoint blockade therapy against CT26 colorectal adenocarcinoma. Results from their study suggested that TBP mediated PDT greatly boosted the *a*-PD-L1 therapy efficacy and elicited an abscopal effect in the treatment of colorectal cancer, which leads to more than 90% regression of tumors. Further mechanistic studies revealed that Fe-TBP plus *a*-PD-L1 treatment significantly induced tumor infiltration of both CD4^+^ and CD8^+^ cytotoxic T cells.

## Current Challenges and Limitations

Over-expressed H_2_O_2_ is produced by the abnormal metabolism of tumor cells (Castaldo et al., 2019), while, targeting CAT to decompose H_2_O_2_ will accelerate the abnormal metabolism of tumor cells remains to be researched. Besides, the expression of CAT in tumor cells is lower than in normal cells (Glorieux et al., 2015), the increasing CAT level in tumor cells may cause changes in the activities of other peroxidases, which may also affect the level of ROS in tumor cells and promoting tumor cell invasion and spreading (Nishikawa et al., 2009). Despite the fabrication of CAT-mimic NCs being very prosperous and obtaining gratifying experimental results, the side effects of metal/metal oxide such as oxidative stress, metabolic alterations, macromolecule dysfunction and cell death need to be further addressed. More importantly, the infiltration of CAT-mimic in tumor tissues has the capability of activating the matrix metalloproteinases, which can cause inflammation and promote tumor metastasis (Matysiak-Kucharek et al., 2018).

Previous research has suggested that very few PFCs are acceptable for parenteral use due to their slow excretion (Cabrales and Intaglietta, 2013). Some commercial PFCs such as Fluosol-DA were withdrawn because of a serious side-effect as well as the unexpected oxygen release. In addition, PFCs can develop an inflammatory reaction when used as a postoperative tamponade for more than one week (Figueroa and Casas, 2014) and intense stromal inflammation when used as a vitreous substitute (Moreira et al., 1992). Besides, PFCs poor water and oil solubility make it hard to modify, which results in limiting its development in tumor therapy. As for Hb, it can also cause severe side effects such as blood clot formation and unacceptable toxicities including renal toxicity and cardiovascular complications in the patient (Jansman and Hosta-Rigau, 2018). Most importantly, PFCs and Hb carrying oxygen would be subject to great losses in blood circulation before reaching the tumor tissue due to blood composition and physiological complexity, which may result in potential oxygen toxicity to organs such as the kidney if the initially carried oxygen is too high.

Compared with traditional drug delivery strategy, the NC drug delivery system is considered to be a promising tool to load anti-tumor drugs and oxygen carriers or generators for tumor therapy owing to their several advantages: 1) increasing half-life of vulnerable drugs and proteins such as CAT; 2) improving the solubility of unstable drugs; 3) allowing surface modified to realize tumor targeting; and 4) achieving sustained and controlled release of drugs in tumors. However, these oxygen-based NCs still have some challenges and limitations, mainly including: 1) the safety of NCs is also not guaranteed and cannot be precisely controlled due to unclear *in vivo* PK process (Zhang A. et al., 2020); 2) the designs of these hypoxia-attenuating NCs in current studies are complicated and are very difficult to translate in large-scale production; and 3) the oxygen released or generated from current NCs in tumor tissues is temporary and small.

## Future Directions

Presently, the approval of both oxygen-generating NCs and oxygen-carrying NCs for enhanced clinical tumor therapy is none. And the translation of tumor oxygen-based NCs from the lab to the clinic is poor. In the end, we propose some of the research directions that should be given more attention in the future, which are based on the challenges and limitations as discussed in the previous section.

Firstly, knowledge regarding the position and degree of hypoxic cells within a tumor could be utilized to improve the personalized and precision medicine. However, existing oxygen measuring methods for tumors are more or less limited by some flaws such as lack of applicability measuring probe and giving only a single time-point measurement. Thus, we should invest more efforts to develop advanced techniques that can accurately and continuously real-time measurement of tumor hypoxia, so that synthesizing the personalized oxygen-based NCs to find better therapeutic modalities by meeting the heterogeneous distribution of tumor hypoxia.

In the second, considering the safety issues of oxygen-based NCs, more attention should be paid to biocompatibility research of NCs and to find safer and more effective oxygen carriers and generators for relieving tumor hypoxia. In addition, it is of great practical importance to master the *in vivo* fate of oxygen-based NCs before reaching the tumor site. And these investigations include, but are not limited to, the types of human solid tumors, the differences of individual such as gender and age, as well as the extrinsic factors such as drug combination on exposure and response. Furthermore, the physicochemical and surface properties of the oxygen-based NCs also should be improved to penetrate tumor tissue and target hypoxic cells at a higher concentration and to reduce the accumulation in non-malignant tissues.

Finally, the production of NCs in the industry not only has to meet the high standards of good manufacturing practices but also needs to be produced at the clinical level in a sustainable manner. Accordingly, the oxygen-based NCs should be designed as simple as possible. What is more, some excellent oxygen-based NCs are designed for intratumoral injection but not systemic injection, which is not best for clinical development. Besides, due to the pathological and oncological differences between mouse-derived tumor-bearing mice and human-derived tumor-bearing mice, it is also of great importance to establish an appropriate preclinical tumor model when involved in the clinical translation of the oxygen-based NCs. In general, humanized mouse tumor models should be more suitable for *in vivo* research.

## Conclusion

Uncontrolled cell proliferation, insufficient blood flow, and inadequate endogenous oxygen lead to tumor hypoxia, which has proven to be one of the primary driving forces for tumor therapy resistance. Replenishing oxygen for alleviating the hypoxic TME is of great importance to improve therapeutic efficacy including CMT, RT, PDT, SDT, and IMT. In this review, we summarized the key oxygen-generating and oxygen-carrying strategies in the field of monotherapies and synergistic combination therapies for tumors. Together with the encouraging results from these studies and rapid advances in biotechnology and oncobiology, we believe that the optimized oxygen-based NCs will continue to grow and may open a new avenue for tumor therapy in the future.

## Glossary

•OH: hydroxyl radical
^1^O_2_: singlet oxygen
APCs: antigen-presenting cells; DCs: dendritic cells
Au_2_O_3_: gold trioxide
BHQ-3: black hole quencher-3
CAT: catalase
CD47: cluster of differentiation 47
Ce: cerium
CMT: chemotherapy
CTLs: cytotoxic T cells;
Cu_2_O: cuprous oxide
CuO: copper oxide
DCs: dendritic cells
DNA: DNA radicals
DOX: doxorubicin
EP: etoposide
EPR: enhanced permeability and retention
ER: endoplasmic reticulum
FA: folate
Fe: iron
H_2_O_2_: hydrogen peroxide
HA: hyaluronic acid
Hb: hemoglobin
HBO: hyperbaric oxygen
HIFs: hypoxia inducible factors
HLA-G: human leukocyte antigen G
HO•: hydroxyl radical
holo-Lf: holo-lactoferrin
HP: hypoxia probe
HSA: human serum albumin
IMT: immunotherapy
MB: methylene blue
MDR1: multidrug resistance gene1
MDSCs: myeloid-derived suppressor cells
Met: metformin
miRNAs: microRNAs
MnCl_2_: manganese chloride
MnO_2_: manganese dioxide
MOF: metal-organic framework
MRI: magnetic resonance imaging
NCs: nanocarriers
NK: natural killer
NPs: nanoparticles
O_2_•‾: superoxide
PB: prussian blue
Pd: palladium
PD-L1: programmed death-ligand 1
PDT: photodynamic therapy
PFCs: perfluorocarbons
PFOB: perfluorooctylbromide
PFP: perfluoropentane
PFTBA: perfluorotributylamine
P-gp: P-glycoprotein
PK: pharmacokinetic
PLGA: Poly(lactic-co-glycolic acid
pO_2_: oxygen partial pressure
Pt: platinum;
PTT: photothermal therapy
PTX: paclitaxel
RBC: red blood cell
RES: reticuloendothelial system
ROS: reactive oxide species
RT: radiotherapy
SDF-1α: stromal cell-derived factor 1α
SDT: sonodynamic therapy
SOR: sorafenib
TAMs: tumor-associated macrophages
TaOx: tantalum oxide
TBP: tetra(p-benzoato)porphyrin
TGI: tumor growth inhibition
TME: tumor microenvironment
Treg: T-regulatory
UCSMs: upconversion nanoprobes
VEGF: vascular endothelial growth factor
WS_2_: tungsten disulfide
ZIF-8: zeolitic imidazolate framework 8
